# Post-Transcatheter Aortic Valve Replacement Antithrombotic Treatment in Nonindicated Patients: Updated Systematic Review and Network Meta-Analysis

**DOI:** 10.1016/j.jacadv.2025.101719

**Published:** 2025-03-31

**Authors:** Sahand Siami, Sina Kazemian, Saba Maleki, Elham Ebrahimi, Fatemeh Jodeiri, Mandana Ebrahimzade, Mohsen Hajiqasemi, Sara Ebrahimi, Maede Mehdizadeh, Mona Aghaei, Mohammad-Mahdi Bastan, Mehrshad Fathian Sabet, Roozbeh Nazari, Pouya Ebrahimi, Jamal S. Rana, Michael G. Nanna, Jay Giri, Dhaval Kolte, Tor Biering-Sørensen, Mohamad Alkhouli, Kaveh Hosseini

**Affiliations:** a Tehran Heart Center, Cardiovascular Diseases Research Institute, Tehran University of Medical Sciences, Tehran, Iran; b School of Medicine, Islamic Azad University, Tehran Medical Branch, Tehran, Iran; c School of Medicine, Shahid Beheshti University of Medical Sciences, Tehran, Iran; d Department of Cardiology, Division of Internal Medicine, The University of Texas MD Anderson Cancer Center, Houston, Texas, USA; e Department of Internal Medicine, McLaren Flint/Michigan State University (MSU), Flint, Michigan, USA; f Cardiovascular Research Center, Shahid Beheshti University of Medical Sciences, Modarres Hospital, Tehran, Iran; g Division of Cardiology, Kaiser Permanente Northern California, Oakland, California, USA; h Division of Research, Kaiser Permanente Northern California, Oakland, California, USA; i Section of Cardiovascular Medicine, Yale University School of Medicine, New Haven, Connecticut, USA; j Cardiovascular Medicine Division, Perelman School of Medicine at the University of Pennsylvania, Philadelphia, Pennsylvania, USA; k Cardiology Division, Massachusetts General Hospital, Boston, Massachusetts, USA; l Harvard Medical School, Boston, Massachusetts, USA; m Department of Cardiology, Herlev and Gentofte University Hospital, Copenhagen, Denmark; n Division of Cardiology, Mayo Clinic Hospital, Rochester, Minnesota, USA

**Keywords:** direct oral anticoagulant, dual antiplatelet therapy, low-dose rivaroxaban, single antiplatelet therapy, transcatheter aortic valve replacement, vitamin K antagonist

## Abstract

**Background:**

The optimal antithrombotic strategy following transcatheter aortic valve replacement (TAVR) remains controversial.

**Objectives:**

The authors aimed to determine the safety and efficacy of various antithrombotic regimens in patients without an indication for anticoagulation following TAVR.

**Methods:**

We conducted a systematic search in PubMed, Embase, Scopus, and ClinicalTrials.gov until August 2024 for studies investigating antithrombotic regimens after TAVR in patients without an indication for chronic oral anticoagulation. The analysis compared single antiplatelet therapy (SAPT), dual antiplatelet therapy (DAPT), direct oral anticoagulants, and oral anticoagulant (OAC) plus SAPT. A frequentist network meta-analysis was employed to evaluate the post-TAVR risk of all-cause mortality, cardiovascular mortality, myocardial infarction, stroke, total bleeding, and life-threatening or major bleeding.

**Results:**

Eleven studies (8 randomized controlled trials and 3 propensity score-matched cohorts) comprising 5,821 patients undergoing TAVR were included. SAPT significantly reduced the risk of life-threatening/major bleeding compared with DAPT (OR: 0.53; 95% CI: 0.35-0.80), OAC (OR: 0.52; 95% CI: 0.28-0.99), and OAC + SAPT (OR: 0.32; 95% CI: 0.13-0.76). No significant differences were observed in the risk of cardiovascular mortality, stroke, or myocardial infarction between antithrombotic regimens. Subgroup analysis indicated an increased risk of mortality with low-dose rivaroxaban+3-month SAPT compared with SAPT (OR: 0.56; 95% CI: 0.35-0.89) and DAPT (OR: 0.58; 95% CI: 0.38-0.88). Meta-regression identified chronic obstructive pulmonary disease as the only significant modifier of bleeding risk following TAVR.

**Conclusions:**

Our findings support current guidelines recommending SAPT as the preferred antithrombotic strategy post-TAVR in patients without an indication for anticoagulation, demonstrating optimal safety without compromising efficacy.

Transcatheter aortic valve replacement (TAVR) has become a minimally invasive and efficient approach for treating symptomatic patients with severe aortic stenosis who were considered high risk for traditional surgery.^1^^,^^2^ However, concerns regarding potential complications persist, ranging from thromboembolic events (eg, valve thrombosis) as well as bleeding caused by post-TAVR treatment to risks of mortality and cardiac death.^1^^,^^3^, ^4^, ^5^ This complexity makes post-TAVR antithrombotic therapy uniquely challenging, mainly as TAVR is currently used for patients with low surgical risk and those with no clear indication for anticoagulation.^2^^,^^6^ Recent evidence has shown that single antiplatelet therapy (SAPT) with aspirin offers more significant benefits than dual antiplatelet therapy (DAPT) with aspirin plus clopidogrel by preventing ischemic complications and reducing the risk of bleeding in patients with no prior need for oral anticoagulants (OACs).^7^, ^8^, ^9^, ^10^, ^11^ The American College of Cardiology (ACC) guidelines recommend lifelong SAPT, preferably aspirin, as a class 2A strategy for post-TAVR patients without anticoagulation indications.^1^^,^^12^ The European Society of Cardiology (ESC) consistently suggests lifelong SAPT with aspirin or clopidogrel with a Class I recommendation for patients without a clear OAC indication.^1^^,^^12^ However, growing concerns about antiplatelet insufficiency in managing post-TAVR valve thrombosis have shifted attention to OACs, particularly vitamin K antagonists (VKA), which have proven more effective than DAPT in preventing valve thrombosis.^1^^,^^13^, ^14^, ^15^ Additionally, several studies have explored the benefits of direct OACs (DOAC) such as edoxaban, apixaban, rivaroxaban, and dual-agent therapy with rivaroxaban plus aspirin following TAVR, especially in reducing the risk of valve thrombosis.^16^, ^17^, ^18^, ^19^, ^20^ While DOACs effectively alleviated the risk of post-TAVR valve thrombosis, they did not show superiority over VKAs or antiplatelets in patients without clinical indications for OAC.^1^^,^^3^^,^^12^^,^^16^ Given the ongoing debates in this field, we aimed to utilize a comprehensive network meta-analysis to determine the safety and efficacy of different post-TAVR antithrombotic therapies in patients without a clear indication for anticoagulation.

## Methods

This systematic review and network meta-analysis adhered to the PRISMA (Preferred Reporting Items for Systematic Reviews and Meta-Analyses) guidelines. The study was also registered in the PROSPERO (International Prospective Register of Systematic Reviews) database (CRD42024587981).

### Search strategy

We conducted a systematic literature search to find studies comparing various antithrombotic regimens in patients undergoing TAVR without a chronic indication for OAC. We searched the PubMed, Scopus, Embase, and ClinicalTrials.gov databases until August 2024. Following the PICO framework, the search strategy focused on 3 components: the population (TAVR patients without an indication for OAC), the intervention (various antithrombotic regimens), and the outcome (major adverse cardiac events [MACE], stroke, mortality, bleeding, and vascular complications). To ensure comprehensive retrieval of relevant studies, medical subject headings (MeSH) terms and keywords for population (eg, “TAVR”), interventions (eg, “SAPT,” “DAPT,” “OAC,” “DOAC,” “VKA”), and outcomes (eg, “total bleeding,” “life-threatening bleeding,” “major bleeding,” “minor bleeding,” “stroke,” “myocardial infarction,” and “cardiovascular mortality”), along with their equivalent keywords. The full search strategy and the queries used for each database can be found in Supplemental Table 1.

#### Eligibility criteria

Studies were included if they matched the following criteria:

• Adult patients who received TAVR without indications for chronic antithrombotic treatment (eg, atrial fibrillation, prosthetic heart valves, cardiovascular interventions).
• Studies with any antithrombotic treatment, including SAPT, DAPT, and OAC consisting of VKA or DOAC and including at least one of the following outcomes: MACE, stroke, all-cause mortality, life-threatening/major bleeding, or vascular complications were included. We included the most recent publication with long-term outcomes for studies with multiple reports. This strategy was selected to ensure the most recent and thorough data.
• Randomized controlled trials (RCTs) and propensity score matched (PSM) cohort studies were considered for inclusion.

Studies including patients already receiving OAC for baseline atrial fibrillation or other indications and those comparing different P2Y12 inhibitor regimens in both case and control arms were excluded. This exclusion criterion was applied to ensure consistency and comparability of control interventions across all included trials. Cohorts without PSM, case reports, reviews, commentaries, and nonhuman studies were also excluded.

### Outcomes

Cardiovascular mortality is defined as death that results directly from cardiovascular causes; this includes death due to myocardial infarction (MI), stroke, heart failure, cardiovascular procedure, bleeding, and sudden cardiac death. Spontaneous MI occurs unrelated to the procedure and is diagnosed by a rise and/or fall of cardiac biomarkers with at least one value above the 99th percentile upper reference limit, along with symptoms of ischemia, new ischemic electrocardiography changes, or evidence of loss of viable myocardium. A stroke was defined as a new, persistent neurological deficit with a variable modified Rankin score that persists for more than 72 hours and causes considerable disability or little to no disability. A brief episode of neurological impairment caused by ischemia (lack of blood flow) without acute infarction (tissue death), with symptoms typically resolving within 24 hours, was also classified as a stroke. Life-threatening bleeding includes fatal bleeding, bleeding in a critical area or organ (eg, intracranial bleeding), bleeding causing hypovolemic shock or requiring emergency surgery, or a significant drop in hemoglobin requiring transfusion of 4 or more units of blood. Major bleeding is defined as bleeding that results in a hemoglobin loss of >3 g/dL, necessitates the transfusion of 2 or more units of blood, or necessitates an extended hospital stay. Any other bleeding that does not meet the mentioned criteria for major and life-threatening bleeding is considered to be minor.^21^ In this study, total bleeding is the composite of life-threatening, disabling, major, and minor bleeding.

### Data extraction

The initial title and abstract screening were divided among 3 groups, each with 2 reviewers. The groups cross-validated each other's work with the supervision of a senior reviewer to ensure accuracy and consistency. For the full-text assessment, 4 independent reviewers retrieved full-text articles from potentially relevant studies and assessed their eligibility. During this phase, discrepancies and conflicts were resolved through discussion and consensus. To ensure accuracy, 2 reviewers cross-checked each article in the final selection. Four reviewers extracted data separately using a standardized data extraction form. The extracted data comprised study characteristics (author, year, study design, sample size, and follow-up duration), patient demographics, details of the antithrombotic regimens, and reported outcomes (MACE, stroke, mortality, bleeding, and vascular complications).

#### Quality assessment

Two reviewers independently evaluated the quality of the included studies using the Cochrane Risk of Bias tool (ROB 2) for RCTs and the Risk of Bias in Non-randomized Studies tool (ROBINS-I) for cohorts with PSM. A third reviewer handled any conflicts during the data extraction or quality evaluation process.

### Statistical analysis

To analyze binary outcomes, we calculated ORs to quantify effect sizes, using random-effect models with the DerSimonian and Laird methods. Heterogeneity was evaluated using Higgins and Thompson's *I*^*2*^ statistic, categorized as follows: *I*^*2*^ <25% indicating low heterogeneity, *I*^*2*^ = 25% to 75% indicating moderate heterogeneity, and *I*^*2*^ >75% indicating high heterogeneity. The between-study variance (*τ*^*2*^) was also assessed, using the Sidik-Jonkman estimator for *τ*^*2*^.

To compare the effectiveness and safety of 4 antithrombotic treatments—SAPT, DAPT, DOACs, and OAC (low-dose rivaroxaban or VKA) + SAPT—we conducted a frequentist network meta-analysis using the *netmeta* package in R. Network consistency was evaluated through node-splitting analysis, which compares direct and indirect evidence. Visual network plots were generated to illustrate direct comparisons between treatments, with line thickness reflecting the number of contributing studies. In subgroup analyses, we separately evaluated the efficacy and safety outcomes by pairwise analyses after dividing the low-dose rivaroxaban + SAPT group from the DOAC and VKA + SAPT groups. Additionally, the VKA and DOAC groups were analyzed independently. Sensitivity analyses were conducted to evaluate the robustness of the results by excluding PSM cohorts and reassessing the primary outcomes.

In addition to the frequentist network meta-analysis, we conducted a Bayesian network meta-analysis to enhance the robustness of our findings and explore the consistency of results under a different statistical framework. We conducted a Bayesian network meta-analysis to compare the effectiveness and safety of antithrombotic treatments—SAPT, DAPT, DOACs, and OAC + SAPT—using the Markov chain Monte Carlo method with 5,000 adaptation iterations followed by 200,000 simulation iterations, assuming transitivity. We estimated ORs with 95% credible intervals (CrIs) based on the medians and 2.5th and 97.5th percentiles of the posterior distributions in a hierarchical Bayesian framework. A random-effects model was used, incorporating informative priors for between-study heterogeneity based on antithrombotic treatments. Convergence was assessed visually using Gelman–Rubin–Brooks plots and statistically using the potential scale reduction factor. Pairwise network comparisons were summarized using forest plots, with SAPT as the reference group. Node-splitting methods were applied to assess inconsistency between direct and indirect evidence.

Meta-regression analyses were performed to evaluate how baseline characteristics, including age, sex, and history of conditions including diabetes, hypertension, MI, peripheral artery disease, chronic obstructive pulmonary disease (COPD), and stroke, the Society of Thoracic Surgeons score, and baseline left ventricular ejection fraction, affected the risk of all-cause mortality and major or life-threatening bleeding outcomes in SAPT vs DAPT. A mixed-effects model was used for this analysis, and the findings were illustrated with bubble plots. To examine potential publication bias, comparison-adjusted funnel plots were visually inspected for symmetry, and Egger's test was applied for statistical confirmation. All statistical analyses were performed using R software (version 4.0.2; R Foundation for Statistical Computing) using the *netmeta*, *meta*, *metafor*, *gemtc*, *rjags*, and *ggplot2* packages. A 2-sided *P* value <0.05 was considered statistically significant.

## Results

**Figure 1:**
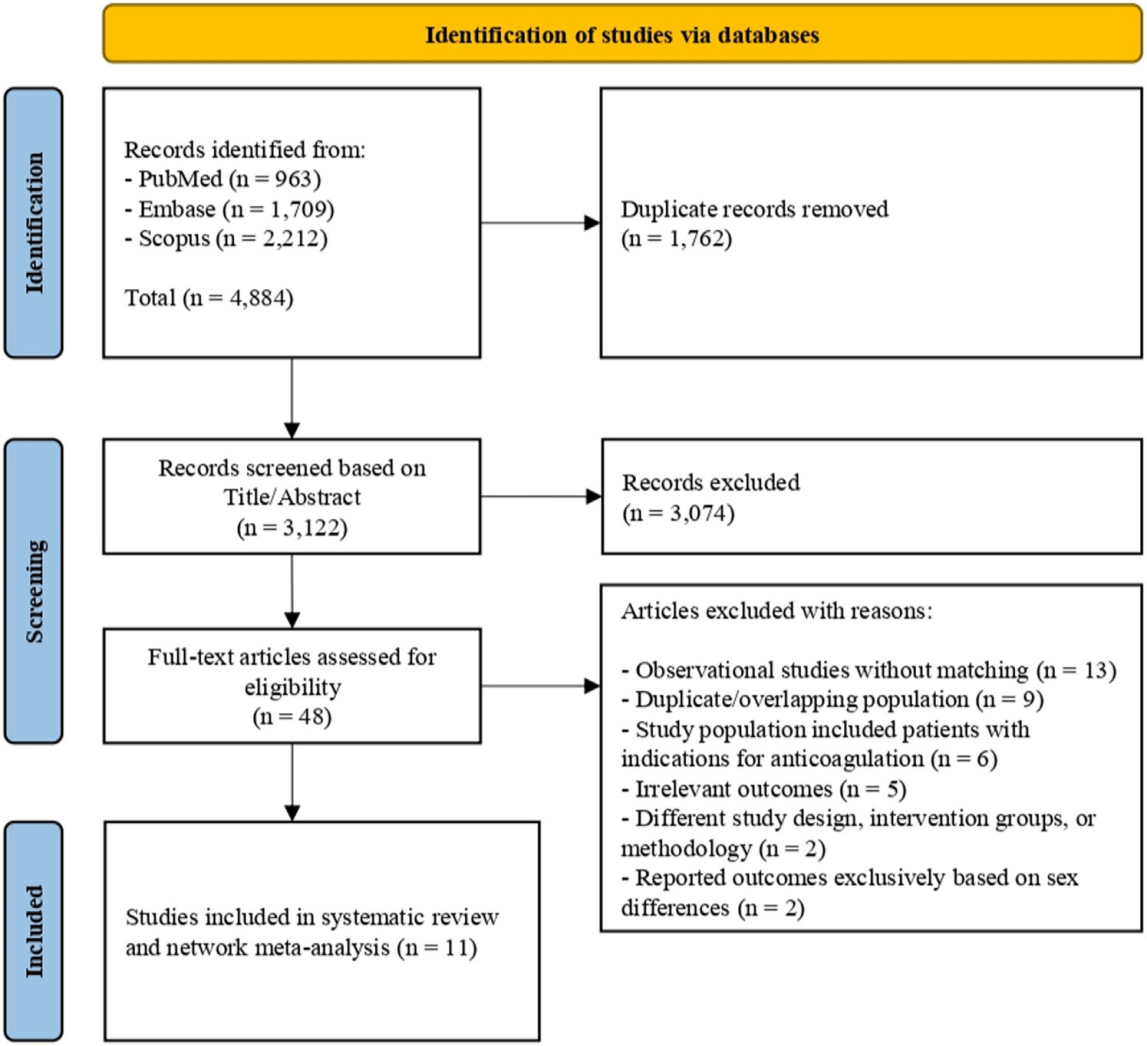
PRISMA Flowchart This flowchart depicts the study selection process following the PRISMA 2020 guidelines, outlining the number of studies identified, screened, assessed for eligibility, and included in the final analysis. PRISMA = Preferred Reporting Items for Systematic Reviews and Meta-Analyses.

**Figure 2:**
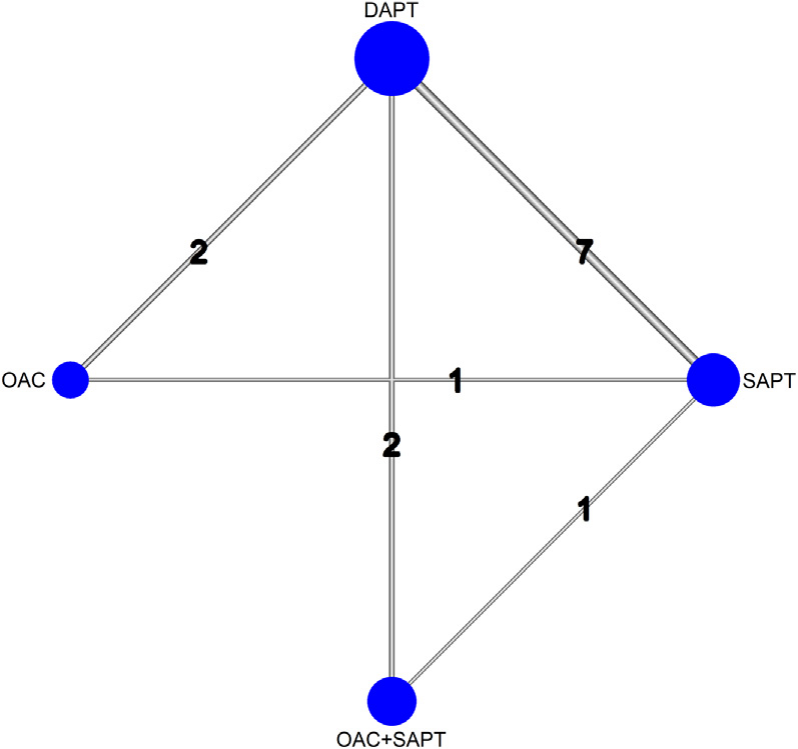
Network Plot of Included Studies This network plot illustrates the comparative evidence for antithrombotic treatment regimens in relation to all-cause mortality. Nodes (circles) represent different treatment strategies, with larger nodes indicating a greater number of patients assigned to that intervention. Edges (lines) denote direct comparisons between treatments, with thicker lines corresponding to a higher number of studies evaluating the respective comparison. DAPT = dual antiplatelet therapy; OAC = oral anticoagulant; SAPT = single antiplatelet therapy.

We identified 4,884 studies from 3 different databases. After removing duplicates and screening the remaining 3,122 studies, we included 8 RCTs^7^^,^^8^^,^^10^^,^^11^^,^^18^, ^19^, ^20^^,^^22^ and 3 PSM cohort studies^23^, ^24^, ^25^ in the final analysis, comprising 5,821 participants undergoing TAVR without indication for chronic anticoagulation treatment (Central Illustration). Details about study selection and screening are presented in Figure 1. Of 5,821 patients, 1,347 (23.2%) were assigned to the SAPT group, 2,694 (46.3%) to the DAPT group, 1,143 (19.6%) to the OAC + SAPT group, and 637 (10.9%) to the OAC group (Figure 2).

**Central Illustration:**
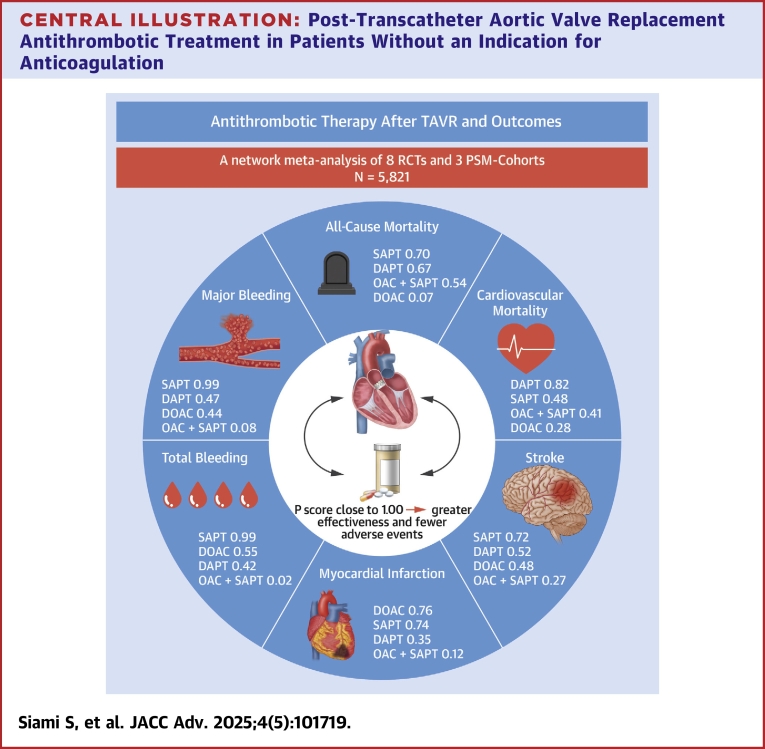
Post-Transcatheter Aortic Valve Replacement Antithrombotic Treatment in Patients Without an Indication for Anticoagulation This central illustration presents a network meta-analysis of 8 RCTs and 3 PSM cohorts (N = 5,821) evaluating the effectiveness and safety of various antithrombotic regimens following TAVR. P scores for all-cause mortality, cardiovascular mortality, stroke, myocardial infarction, major bleeding, and total bleeding are shown for each treatment regimen. A P score closer to 1.00 indicates greater effectiveness and fewer adverse events. DOAC = direct oral anticoagulant; PSM = propensity score-matched; RCT = randomized controlled trial; other abbreviations as in Figures 2, 3, and 5.

Among the 5,821 participants, the mean age was 80.7 ± 6.5 years, 51.7% were female, and the mean Society of Thoracic Surgeons risk score was 5.5 ± 4.8 (Table 1). We observed that 81.3% and 28.5% of participants had a history of hypertension and diabetes mellitus, respectively. Balloon-expandable valves (BEVs) were the most predominant reported valve types as they were used in 2,805 (69.9%) patients, followed by self-expandable valves, which were used in 1,210 (30.1%) patients. The baseline characteristics of the participants in each study are reported in Supplemental Tables 1 and 2.

**Table 1:** Study Characteristics

First Author, Year	Study Design	No. of Participants			Antithrombotic Regimen Following TAVR		Duration of Antithrombotic Treatment Following TAVR		Outcomes	Follow-Up Duration
		Total	Arm 1	Arm 2	Arm 1	Arm 2	Arm 1	Arm 2		
SAPT vs DAPT										
Ussia et al, 2011^7^	Open-label single-center RCT	79	39	40	Aspirin only (100 mg, daily) (SAPT)	LD = 300 mg clopidogrel Aspirin (100 mg, daily) + clopidogrel (75 mg, daily) (DAPT)	SAPT for the duration of the trial and then lifelong	DAPT for the initial 3 mo, followed by SAPT lifelong	The composite of MACE including all-cause death, MI, major stroke, urgent or emergency conversion to surgery, and life-threatening bleeding	6 mo
Stabile et al, 2014^11^	Pilot randomized single-center trial	120	60	60	Aspirin only (75-160 mg, daily) (SAPT)	Aspirin (75-160 mg, daily) + clopidogrel (75 mg, daily) or ticlopidine (500 mg, twice a day) (DAPT)	SAPT for the duration of the trial and then lifelong	DAPT for the initial 6 mo, followed by SAPT lifelong	All-cause mortality, VARC-defined endpoints	6 mo
D'Ascenzo et al, 2017^23^	Retrospective PSM analysis from ITER Registry	1210 (post-PSM)	605	605	Aspirin only (80 mg, daily) (SAPT)	Aspirin (80-100 mg, daily) + clopidogrel (75 mg, daily) (DAPT)	6 mo	6 mo	Prosthetic heart valve dysfunction at follow-up	14 mo
Ichibori et al, 2017^24^	Nonrandomized retrospective cohort with PSM analysis	88	44	44	Aspirin only (80-100 mg, daily) (SAPT)	Aspirin (80-100 mg, daily) + clopidogrel (75 mg, daily) + ticlopidine (DAPT)	12 mo	12 mo	All-cause death, MI, stroke, and major or life-threatening bleeding complications	12 mo
Rodés-Cabau et al, 2017^8^	Open-label multicenter RCT	222 (as-treated = 219)	111 (as-treated = 109)	111 (as-treated = 110)	Aspirin only (80-100 mg, daily) (SAPT)	LD = 300 mg clopidogrel Aspirin (80-100 mg, daily) + clopidogrel (75 mg, daily) (DAPT)	SAPT continued for at least 6 mo	DAPT for the initial 3 mo, followed by SAPT for at least 6 mo	The rate of death, MI, ischemic stroke or TIA, or major or life-threatening bleeding	3 mo
Brouwer et al, 2020^10^	Open-label multicenter RCT	690 (modified ITT = 665)	331	334	LD = 300 mg aspirin Aspirin only (80-100 mg, daily) (SAPT)	LD = 300 mg aspirin + 300 mg clopidogrel Aspirin (80-100 mg, daily) + clopidogrel (75 mg, daily) (DAPT)	SAPT for the duration of the trial and then lifelong	DAPT for the initial 3 mo, followed by SAPT	All bleeding, nonprocedure-related bleeding	12 mo
SAPT vs OAC + SAPT										
Merdler et al 2023^a^^,^^b^^,^^22^	Open-label multicenter RCT	94	50	44	Low-dose aspirin (81-100 mg, daily) (SAPT)	Low-dose aspirin + warfarin	SAPT for 1 mo, followed by ATR upon physician discretion	Warfarin + SAPT for 1 mo, followed by ATR upon physician discretion	All-cause mortality, HALT, at least moderate RELM, hemodynamic dysfunction, stroke, TIA, life-threatening and major bleeding, major vascular complications, AKI, pacemaker implantation	24 mo
DAPT vs OAC										
Park et al, 2022^18^	Open-label multicenter RCT	229	118	111	Aspirin (100 mg, daily) + clopidogrel (75 mg, daily) (DAPT)	Edoxaban (30-60 mg, daily)	DAPT for 6 mo	Edoxaban for 6 mo	The incidence of valve leaflet thrombosis	6 mo
DAPT vs OAC + SAPT										
Dangas et al, 2020^19^	Open-label multicenter RCT	1,644	818	826	LD = 300 mg clopidogrel Aspirin (75-100 mg, daily) + clopidogrel (75 mg, daily) (DAPT)	Rivaroxaban (10 mg, daily) + aspirin (75-100 mg, daily) (SAPT)	DAPT for 3 mo, followed by SAPT	Rivaroxaban + SAPT for 3 mo, followed by rivaroxaban	The composite of all-cause death or TE (stroke, MI, symptomatic valve thrombosis, noncentral nervous system SE, DVT, or PE), life-threatening, disabling, or major bleeding	17 mo
Collet et al, 2022^c^^,^^20^	Open-label multicenter RCT	1,047	521	526	SAPT or DAPT with aspirin and clopidogrel (doses upon physician discretion)	Apixaban (2.5-5 mg, twice a day) or apixaban + SAPT or triple therapy with aspirin and clopidogrel	Not specified, but in the case of DAPT, 6 mo was applied	Apixaban for 12 mo	The composite of death, MI, stroke or TIA, noncentral nervous system SE, intracardiac or valve thrombosis, episode of DVT or PE, life-threatening, disabling, or major bleeding	12 mo
Naser et al 2023^25^	Retrospective cohort with PSM	426	153	273	Aspirin + clopidogrel (DAPT)	VKA (warfarin) + SAPT	3-6 mo	3 mo + SAPT	Evaluation of the medium-term outcomes of ischemic stroke, death, valve replacement or intervention, and structural valve degeneration	2.7 (1.1-4.2)

AKI = acute kidney injury; ATR = antithrombotic regimen; DAPT = dual antiplatelet therapy; DVT = deep vein thrombosis; HALT = hypoattenuated leaflet thickening; ITER = Italian Transcatheter Balloon-Expandable Valve Implantation Registry; ITT = intention-to-treat; LD = loading dose; MACE = major adverse cardiac events; MI = myocardial infarction; OAC = oral anticoagulant; PE = pulmonary embolism; PSM = propensity score matching; RCT = randomized controlled trial; RELM = reduced leaflet motion; SAPT = single antiplatelet therapy; SE = systemic embolism; TAVR = transcatheter aortic valve replacement; TE = thromboembolic events; TIA = transient ischemic attack; VARC = Valve Academic Research Consortium; VKA = vitamin K antagonist.

a The study by Rogers et al (2021)^15^ is a 30-day follow-up, while the study by Merdler et al (2023)^22^ is a 24-month follow-up survey of LRT 2.0, both using the same study population for analysis.

b In both studies by Rogers et al (2021)^15^ and Merdler et al (2023),^22^ all patients received VKA plus SAPT for 30 days, with VKA continuation beyond 30 days left to the physician's discretion.

c In the study by Collet et al (2022)^20^ (Stratum 2), the DOAC arm included 3 regimens: 57.8% received apixaban alone, 35.5% received apixaban + SAPT, and 6.7% received triple therapy with apixaban + DAPT.

### Efficacy outcomes

**Figure 3:**
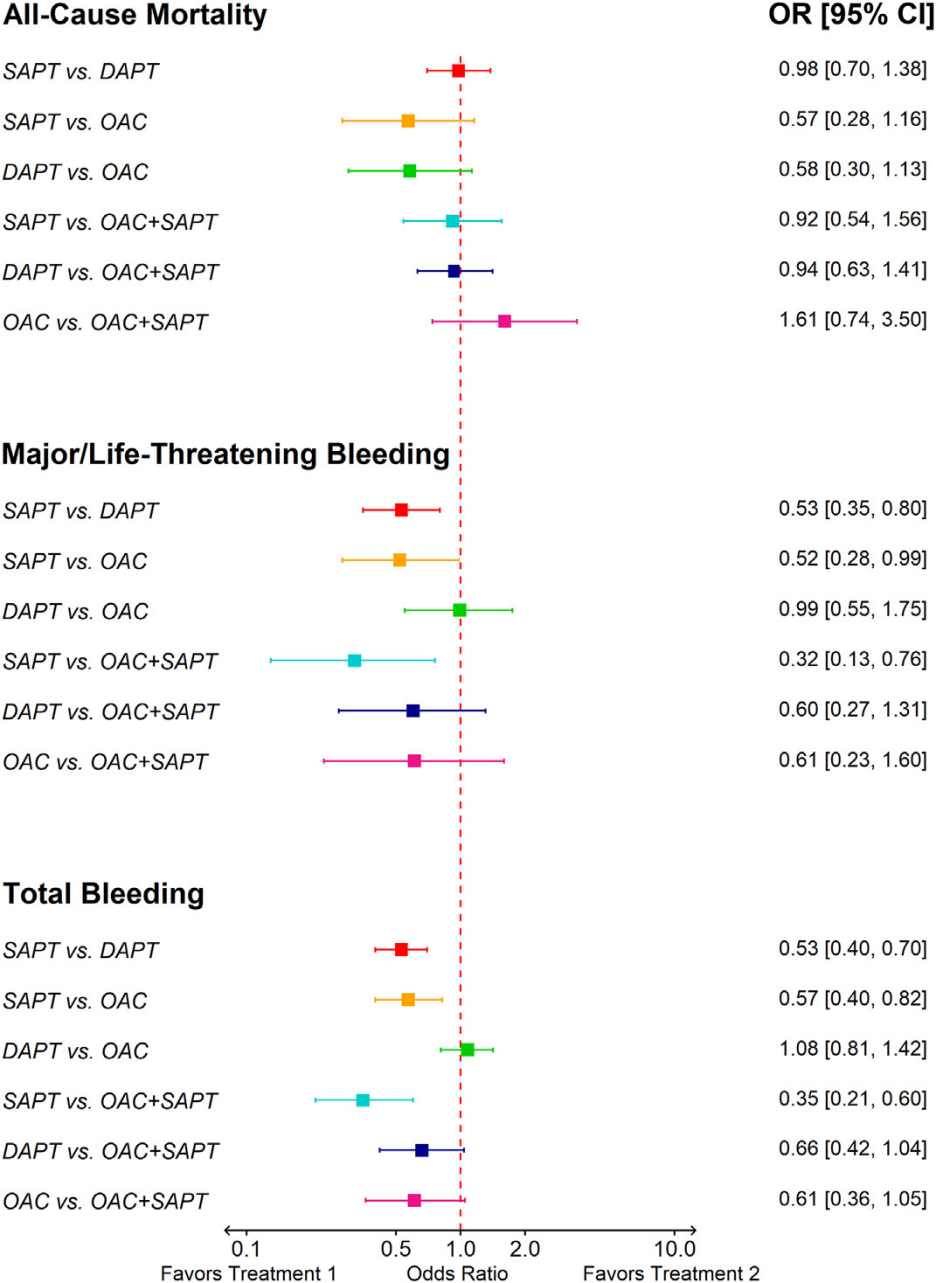
Pairwise Network Meta-Analysis of Primary Efficacy and Safety Outcomes This figure presents a pairwise network meta-analysis comparing SAPT, DAPT, OAC, and OAC + SAPT for all-cause mortality, major or life-threatening bleeding, and total bleeding. ORs with 95% CIs are displayed for each comparison. OAC + SAPT = oral anticoagulant plus single antiplatelet therapy; other abbreviations as in Figure 2.

After a mean follow-up duration of 18.0 ± 4.8 months (ranging from 3 to 31 months), we observed the following results for each treatment comparison across key efficacy outcomes: The network estimate did not show any significant difference in all-cause mortality in SAPT compared with DAPT. The network estimate did not indicate any significant difference in the risk of all-cause mortality but a trend favoring DAPT compared with OAC (OR: 0.58; 95% CI: 0.30-1.13; *P* = 0.11). The network estimate for SAPT vs OAC did not suggest any significant difference in the risk of all-cause mortality but a trend favoring SAPT (OR: 0.57; 95% CI: 0.28-1.16; *P* = 0.12) (Figure 3). SAPT ranked as the best treatment for preventing all-cause mortality following TAVR (P score = 0.70) (Table 2). The *I*^*2*^ values in direct estimates were generally low (<25%), with most direct comparisons showing 0% heterogeneity. The DAPT vs OAC + SAPT comparison demonstrated high heterogeneity (*I*^*2*^: 90%), which suggests inconsistency in the results for this particular comparison across studies (Supplemental Figure 1).

**Table 2:** Ranking of Antithrombotic Regimens for Safety and Efficacy Outcomes Based on P Score

Treatment	All-Cause Mortality	Cardiovascular Mortality	Total Bleeding	Major or Life-Threatening Bleeding	Myocardial Infarction	Stroke
SAPT	0.70	0.48	0.99	0.99	0.74	0.72
DOAC	0.07	0.28	0.55	0.44	0.76	0.48
DAPT	0.67	0.82	0.42	0.47	0.35	0.52
OAC + SAPT	0.54	0.41	0.02	0.08	0.12	0.27

The P score indicates the relative superiority of each treatment. A P score closer to 1.00 suggests greater efficacy and lower adverse event risk.

DOAC = direct oral anticoagulant; other abbreviations as in Table 1.

**Figure 4:**
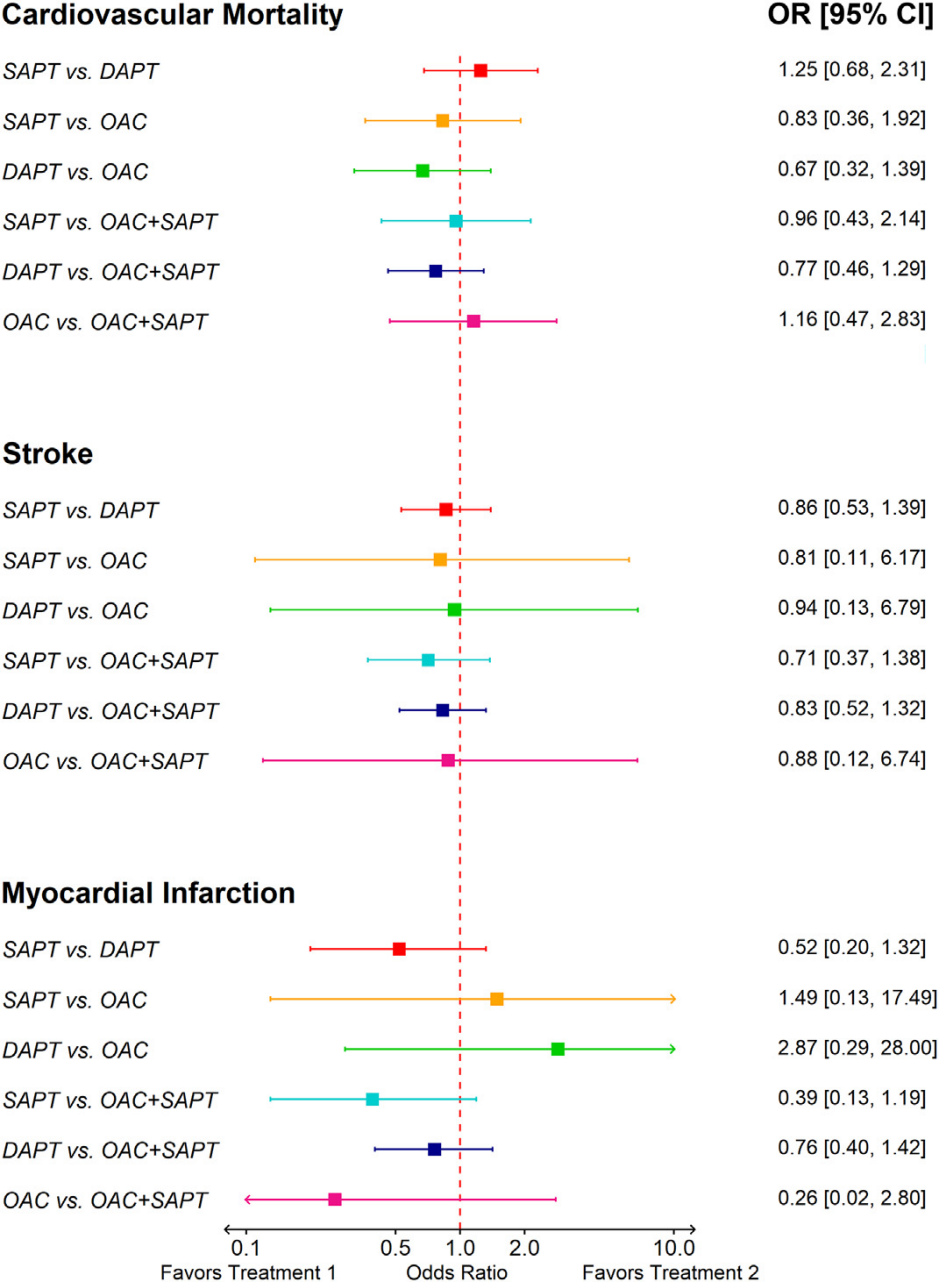
Pairwise Network Meta-Analysis of Secondary Efficacy and Safety Outcomes This figure presents a pairwise network meta-analysis comparing SAPT, DAPT, OAC, and OAC + SAPT for cardiovascular mortality, stroke, and myocardial infarction. ORs with 95% CIs are displayed for each comparison. Abbreviations as in Figures 2 and 3.

The network estimate suggests that there is no significant difference in cardiovascular mortality between pairwise groups (SAPT vs DAPT: OR: 1.25; 95% CI: 0.68-2.31; *P* = 0.47; DAPT vs OAC: OR: 0.67; 95% CI: 0.32-1.39; *P* = 0.28; and SAPT vs OAC: OR: 0.83; 95% CI: 0.36-1.92; *P* = 0.66) (Figure 4). DAPT ranked as the best treatment for preventing cardiovascular mortality following TAVR (P score = 0.82) (Table 2).

### Safety outcomes

SAPT was associated with a significant reduction in both risk of major or life-threatening and total bleeding compared with DAPT (OR: 0.53; 95% CI: 0.35-0.80; *P* = 0.003 and OR: 0.53; 95% CI: 0.40-0.70; *P* < 0.001, respectively), OAC (OR: 0.52; 95% CI: 0.28-0.99; *P* = 0.042 and OR: 0.57; 95% CI: 0.40-0.82; *P* = 0.002, respectively), and OAC + SAPT (OR: 0.32; 95% CI: 0.13-0.76; *P* = 0.011 and OR: 0.35; 95% CI: 0.21-0.60; *P* < 0.001, respectively). However, DAPT did not reduce the risk of major or life-threatening bleeding and total bleeding significantly compared with OAC (OR: 0.99; 95% CI: 0.55-1.75; *P* = 0.97 and OR: 1.08; 95% CI: 0.81-1.42; *P* = 0.59, respectively) OAC or OAC + SAPT (OR: 0.60; 95% CI: 0.27-1.31; *P* = 0.20 and OR: 0.66; 95% CI: 0.42-1.04; *P* = 0.072, respectively). However, total bleeding showed a trend favoring OAC. Additionally, adding SAPT to OAC did not cause a significant increase in the risk of major or life-threatening bleeding (OR: 0.61; 95% CI: 0.23-1.60; *P* = 0.32) or total bleeding (OR: 0.61; 95% CI: 0.36-1.05; *P* = 0.070) compared with OAC alone (Figure 3, Supplemental Figure 1). SAPT ranked as the best treatment for prevention of bleeding following TAVR (P score = 0.99) (Table 2).

The risk of MI and stroke did not differ significantly between various regimens (Figure 4). However, based on P scores, DOAC and SAPT ranked as the best antithrombotic regimens for preventing MI and stroke following TAVR, respectively (DOAC P score = 0.76 and SAPT P score = 0.72) (Table 2). The between- and within-study heterogeneity was low (*I*^*2*^ <25% and Cochran's Q test *P* > 0.05) for all safety outcomes. Moreover, network node-splitting analysis showed no statistical inconsistency (Supplemental Figure 1). More details on the number of clinical and imaging events in the participants of each trial were reported in Supplemental Table 3.

### Meta-regression

**Figure 5:**
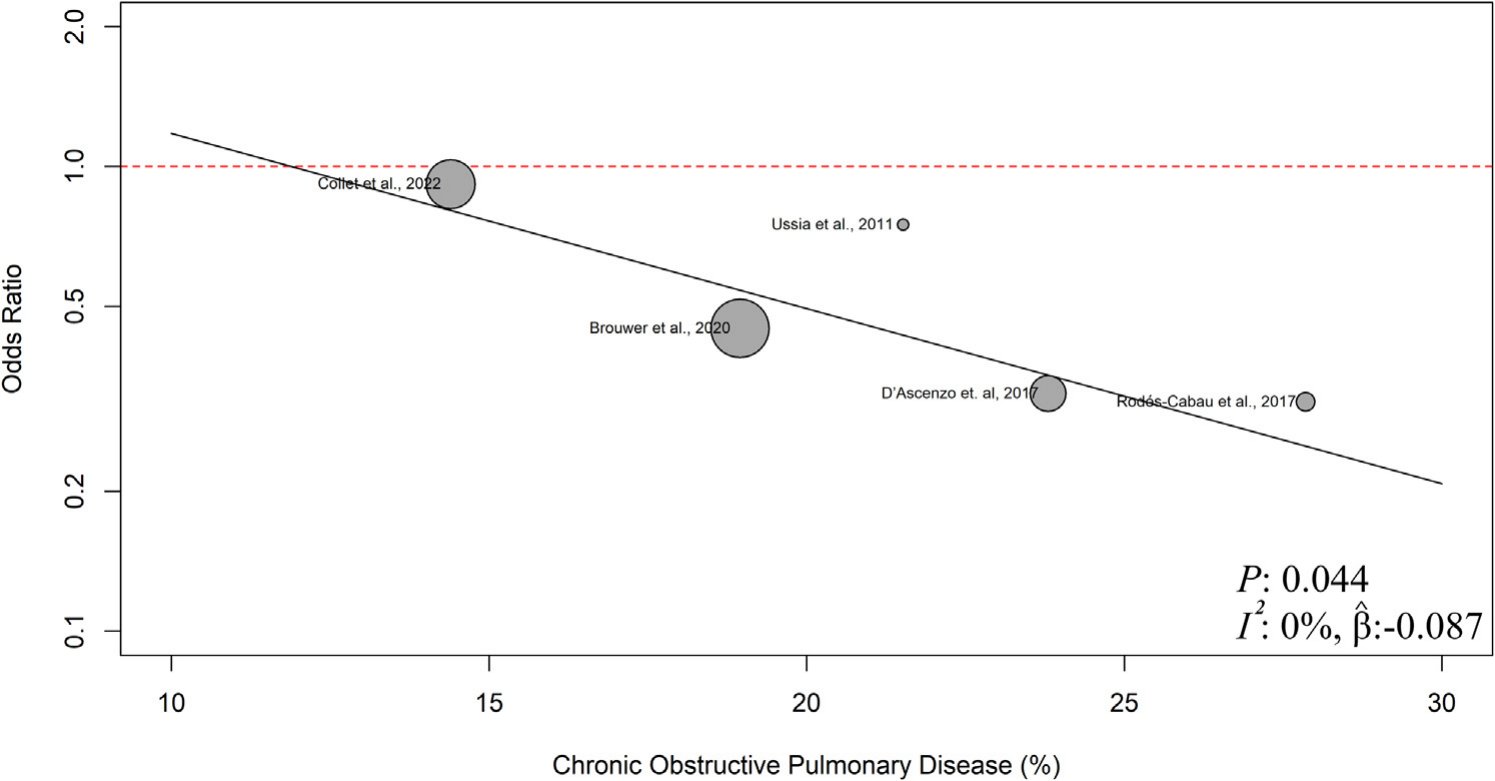
Bubble Plot of Meta-Regression Analysis on Chronic Obstructive Pulmonary Disease This bubble plot illustrates the meta-regression analysis assessing the modifying effect of COPD on the risk of major or life-threatening bleeding in patients receiving SAPT vs DAPT following TAVR. The regression line represents the relationship between COPD prevalence and the OR for major or life-threatening bleeding with SAPT compared with DAPT, while the bubble size corresponds to the study's weight in the analysis. COPD = chronic obstructive pulmonary disease; TAVR = transcatheter aortic valve replacement; other abbreviations as in Figure 2.

Meta-regression analyses were conducted to examine the modifying effects of patients' baseline characteristics on primary safety and efficacy outcomes. Our analyses revealed that COPD was the only factor with a significant modifying effect on the reduced risk of major or life-threatening bleeding after SAPT vs DAPT, with an estimated regression coefficient ($\hat{\beta }$) of 0.087, indicating the change in the log OR for the outcome per increase in COPD percentage (*P* = 0.044; *I*^*2*^ = 0%) (Figure 5). No other baseline characteristics showed a significant modifying effect on the risk of major or life-threatening bleeding or all-cause mortality. The bubble plots of each baseline characteristic are provided in the Supplemental Figure 2.

### Subgroup analysis

#### Separating low-dose rivaroxaban+3-month SAPT from other regimens

In a subgroup analysis, we considered low-dose rivaroxaban+3-month SAPT as a separate treatment arm to understand the specific impact of low-dose rivaroxaban vs other treatments, including SAPT, DAPT, and VKA. Both SAPT and DAPT showed a significant reduction in all-cause mortality compared with low-dose rivaroxaban+3-month SAPT (OR: 0.56; 95% CI: 0.35-0.89; *P* = 0.015 and OR: 0.58; 95% CI: 0.38-0.88; *P* = 0.011, respectively). Also, low-dose rivaroxaban+3-month SAPT was associated with a significant increase in the risk of all-cause mortality compared with VKA + SAPT (OR: 2.55; 95% CI: 1.44-4.52; *P* = 0.001). SAPT was associated with a reduced risk of major or life-threatening bleeding and total bleeding compared with low-dose rivaroxaban+3-month SAPT (OR: 0.25; 95% CI: 0.11-0.57; *P* = 0.001 and OR: 0.35; 95% CI: 0.21-0.60; *P* < 0.001, respectively). No significant differences were observed in the risk of cardiovascular mortality, MI, or stroke between low-dose rivaroxaban+3-month SAPT and other antithrombotic regimens (Supplemental Figure 3).

#### Comparison of DOAC with or without SAPT with other regimens

In this subgroup analysis, DOAC with or without SAPT (DOAC *±* SAPT) and VKA + SAPT were separated instead of being considered as DOAC and OAC + SAPT groups in the main analysis. This differentiation allowed for a more precise comparison between the effects of DOAC and VKA + SAPT. The DOAC *±* SAPT was associated with a significantly increased risk of all-cause mortality compared with VKA + SAPT. Additionally, DOAC ± SAPT was associated with a higher risk of major bleeding compared with VKA + SAPT. No significant difference was observed between DOAC ± SAPT and VKA + SAPT in terms of stroke or MI risk (Supplemental Figure 4).

#### Sensitivity analysis after removing PSM cohort studies

In this sensitivity analysis, we removed 3 PSM studies from the total data set to repeat the analysis only on RCTs. The objective was to evaluate the robustness of the results and examine how excluding these studies impacted the findings. After excluding the PSM cohorts from the included studies, DAPT was associated with a significant reduction in all-cause mortality compared with OAC + SAPT (OR: 0.58; 95% CI: 0.39-0.88; *P* = 0.009). Furthermore, SAPT continued to show no significant difference compared to DAPT or DOAC regarding the risk of all-cause mortality. The exclusion of PSM studies reinforced the earlier findings that SAPT was associated with a significantly reduced risk of major or life-threatening bleeding compared with both DAPT and OAC + SAPT (Supplemental Figure 5). Similar to the main analysis, no significant differences were found in the risk of cardiovascular mortality across antithrombotic regimens, indicating the robustness of our findings even after removing PSM studies (Supplemental Figure 6).

#### Bayesian network meta-analysis

The Bayesian network meta-analysis indicated no significant difference in the risk of all-cause mortality, cardiovascular mortality, MI, and stroke between antithrombotic regimens following TAVR (Supplemental Figure 7). Compared with SAPT, DAPT was associated with a higher risk of major or life-threatening bleeding (OR: 1.9; 95% CrI: 1.1-3.3) (Supplemental Figure 7B) and total bleeding (OR: 1.9; 95% CrI: 1.2-2.8) (Supplemental Figure 7C). Additionally, OAC + SAPT was associated with an increased risk of total bleeding compared with SAPT (OR: 2.8; 95% CrI: 1.1-7.1). There was no significant difference in the risk of total bleeding or major or life-threatening bleeding between other antithrombotic regimens. The potential scale reduction factor indicated good convergence across all outcomes (Supplemental Figure 7). Node-splitting analysis demonstrated no evidence of network inconsistency between direct vs indirect evidence (Supplemental Figure 8).

#### Publication bias

The visual assessment of the funnel plots and Egger's test indicated no evidence of publication bias for all-cause mortality (*P* = 0.95), cardiovascular mortality (*P* = 0.33), total bleeding (*P* = 0.72), major or life-threatening bleeding (*P* = 0.34), MI (*P* = 0.46), and stroke (*P* = 0.99). Furthermore, Begg's test indicated no evidence of publication bias across all outcomes in the comparison between SAPT and DAPT (Supplemental Table 4). Funnel plots for each outcome are provided in Supplemental Figure 7.

#### Quality assessment

The quality of RCTs was assessed using the RoB-2 quality assessment tool. We found that 6 (75%) had a low risk of bias or some concerns, although 2 RCTs were identified as having a high risk of bias (Supplemental Figure 8). All PSM cohorts were evaluated as having a moderate risk of bias based on the ROBINS-I tool (Supplemental Figure 9).

## Discussion

The current network meta-analysis depicted SAPT as a prudent post-TAVR antithrombotic strategy with the most balance between safety and efficacy in patients with no indication for long-term OAC therapy. Postprocedural SAPT significantly reduced safety outcomes of total and major/life-threatening bleeding compared with DAPT, DOAC, or OAC + SAPT. Meanwhile, there were no differences in terms of cardiovascular mortality, stroke, and MI across all regimens. Although no significant difference in all-cause mortality was retrieved among the 4 main arms of the study, there was a trend against DOAC ± SAPT (driven by low-dose rivaroxaban+3-month SAPT) in sensitivity analyses compared with SAPT, DAPT, and even VKA + SAPT.

Contemporary guidelines—ACC/American Heart Association 2020 (Class 2A) and ESC/European Association for Cardio-Thoracic Surgery 2021 (Class І)—recommend lifelong SAPT for patients with bioprosthetic TAVR who have no other baseline indications for OAC.^26^^,^^27^ This statement supports our findings that SAPT is the safest and most effective approach, as it lowers the risk of bleeding and stroke and reduces all-cause mortality. Besides, our initial findings of a significant reduction of major or life-threatening bleeding due to SAPT compared to DAPT and OAC + SAPT were fortified by their consistency in our sensitivity analyses. We must highlight that these findings should be interpreted with extra caution, as some pairwise analysis results are predominantly based on indirect evidence, which may introduce uncertainty and limit external validity. Therefore, further randomized trials are needed to confirm these results. In alignment with our study, a similar network meta-analysis of 7 RCTs by Guedeney et al^28^ demonstrated that SAPT more than halved the major or life-threatening bleeding compared with DAPT or DOAC ± SAPT without significant difference in terms of ischemic complications including MI, stroke, and systemic embolism. Turgeon et al's relevant network meta-analysis of 11 RCTs also stated both SAPT and DAPT as the best-ranking treatments in decreasing all-cause death and MACE compared to DOAC, VKA, or OAC + SAPT. At the same time, SAPT ranked first for major bleeding.^29^ The rationale for SAPT's superior risk-benefit profile is rooted in the prevention of thromboembolic events as effectively as DAPT or anticoagulant-based regimens, without imposing an additional risk of bleeding, especially in older adults suffering from multiple comorbidities who encounter a serious concern of hemorrhagic complications.

Furthermore, reviewing P score values depicted the most efficient regimen to be DAPT in cardiovascular mortality, SAPT in stroke, and DOAC in MI. However, statistical significance was not retrieved through pairwise comparisons regarding these 3 outcomes, which aligned with the previous articles.^28^, ^29^, ^30^

Although none of the comparisons for all-cause mortality were significantly different among various regimens, a trend favoring antiplatelets was shown compared to DOAC. Further separation of DOAC ± SAPT and VKA + SAPT revealed a significantly higher risk of all-cause mortality with DOAC ± SAPT compared to antiplatelets and VKA + SAPT, primarily driven by low-dose rivaroxaban+3-month SAPT. In line with our findings, Guedeney et al^23^ showed a significant reduction of all-cause mortality by DAPT vs low-dose rivaroxaban + SAPT. Our sensitivity analysis of RCTs, excluding PSM cohorts, also indicated that DAPT was the only regimen to show a statistically significant benefit in reducing all-cause mortality when compared to OAC + SAPT, supporting Turgeon et al's conclusion.^29^ Nevertheless, the mentioned study^29^ did not differentiate between the types of anticoagulants combined with SAPT and their variations in dosage, as their OAC + SAPT group represented the addition of SAPT to either a standard dose of VKA or a low dose of rivaroxaban (10 mg daily). Besides, the trials with patients having anticoagulation indications were included in this study, diminishing the reliability of the study's implication compared to our only “no indication” setting. Even though the other network analysis by Guedeney et al^28^ noticed the variation of dosage in DOACs and the association of low-dose rivaroxaban + SAPT with a higher risk of all-cause mortality as well as noncardiovascular mortality, VKA + SAPT regimen was still not considered in this study, making our network meta-analysis the most comprehensive one in patients with no anticoagulation indications. Accordingly, ACC/American Heart Association 2020 guidelines have contraindicated (Class: Harm) the addition of low-dose rivaroxaban (10 mg daily) to aspirin for patients with bioprosthetic transcatheter aortic valve in the absence of other indications for OAC.^26^ In line with this guideline, our study found that combining VKA with SAPT appeared to be a more effective treatment than low-dose rivaroxaban with 3-month SAPT, as it led to significantly lower rates of both all-cause mortality and major bleeding. Additionally, we observed a trend favoring VKA + SAPT over SAPT or DAPT in these patients, though the wide confidence intervals indicate that further research is needed to confirm these findings. Despite DOACs' better protection against reduced leaflet motion, hypoattenuating leaflet thickening,^28^^,^^31^ or valve thrombosis^30^^,^^32^ at standard dosage, compared with antiplatelet therapy, they have been denounced by some meta-analyses due to their association with a higher incidence of all-cause mortality probably due to non-CV causes.^31^, ^32^, ^33^ A trend against DOAC vs SAPT or DAPT in all-cause mortality was also obtained when we separated other DOACs from low-dose rivaroxaban+3-month SAPT. However, it failed to reach statistical significance.

The meta-regression analysis revealed that the decreased risk of major or life-threatening bleeding with SAPT was significantly intensified compared to DAPT as the prevalence of COPD rose. This study is the first to use meta-regression analysis to evaluate how baseline characteristics, such as COPD, modify cardiovascular outcomes following different post-TAVR antithrombotic strategies. Although a pair of included studies^23^^,^^24^ sought the possible predictive factors of antithrombotic outcomes, COPD had not been entered in their regression analyses. Our findings suggest that SAPT may be a safer antithrombotic than DAPT in patients with COPD, given its more significant reduction in the risk of major or life-threatening bleeding. As COPD patients are associated with a higher risk of post-TAVR late bleeding events, identifying the best antithrombotic regimen for preventing bleeding events in this population is crucial.^34^ Moreover, the impact of COPD on TAVR outcomes remains unclear and should be further investigated in future trials.

Our Bayesian network meta-analysis largely confirmed the findings of the frequentist approach, demonstrating consistency across all primary efficacy outcomes. However, differences emerged in bleeding risk estimates, where the frequentist model indicated a significantly higher risk of total bleeding and major or life-threatening bleeding for OAC vs SAPT and an increased risk of major/life-threatening bleeding for OAC + SAPT vs SAPT, while these associations became nonsignificant in the Bayesian model. This discrepancy can be attributed to the limited direct evidence (1 study per comparison) and greater reliance on indirect comparisons, leading to wider credible intervals and Bayesian shrinkage effects. These findings highlight the need for future randomized trials to provide more robust direct evidence to compare the risk of bleeding after OAC and OAC + SAPT vs SAPT in patients without indication for anticoagulation undergoing TAVR.

Although no analysis was conducted regarding antithrombotic regimen duration in our study, a comprehensive review of each study's results highlighted conclusions similar to those of Kuno et al,^35^ as no differences were observed between 3-month and 6-month DAPT in terms of safety and efficacy outcomes. Additionally, for OACs, there appeared to be no apparent differences in outcomes between OACs taken for 3 months or less and those taken for more than 3 months, which may be influenced by the type of OAC (VKA vs DOAC) rather than duration alone.

### Limitations and future directions

Our meta-analysis has several limitations that need consideration. First, variations in study design, follow-up duration, and treatment protocols introduce between-study heterogeneity. Differences in antithrombotic regimen and OAC dosages across studies further contribute to variability. Most studies reported outcomes at inconsistent follow-up time points, requiring data extraction from the latest available period in each study. Second, the limited availability of direct comparisons for “OAC” and “OAC + SAPT” pairwise analyses necessitated reliance on predominantly indirect evidence, which may impact the generalizability and external validity of our findings. However, our network meta-analysis demonstrated no significant heterogeneity or inconsistency, reinforcing the credibility of our results despite sparse direct comparisons. Importantly, network meta-analysis is designed to integrate limited direct evidence with indirect comparisons, making it an optimal methodological approach for research settings where head-to-head trials are sparse. Third, periprocedural effects of antithrombotic regimens were not evaluated, potentially leading to an underestimation of their long-term impact. Fourth, new-onset atrial fibrillation was pooled with preexisting atrial fibrillation in baseline characteristics, preventing an independent analysis of its impact. Further studies are needed to assess new-onset atrial fibrillation as a distinct outcome to enhance understanding of its association with different antithrombotic strategies. Fifth, we could not assess the association between postprocedural subclinical outcomes such as hypoattenuating leaflet thickening and reduced leaflet motion because of a lack of data in the included studies. Lastly, 69.9% of the studies included in our analysis used BEVs, which limited our ability to conduct a comprehensive comparative analysis based on aortic valve type or generation. Future research should investigate potential differences between BEVs and self-expandable valves to refine treatment recommendations.

## Conclusions

This comprehensive network meta-analysis of 11 studies (8 RCTs and 3 PSM cohorts) comparing various antithrombotic strategies, recommends SAPT as the safest antithrombotic therapy for patients without an indication for anticoagulation following TAVR. SAPT ranked as the best treatment option for prevention of all-cause mortality with the lowest risk of bleeding. At the same time, our analyses suggested a trend toward higher all-cause mortality in patients taking DOACs compared with antiplatelets following TAVR. Further studies are needed to assess the safety and efficacy of DOACs and VKAs after TAVR in patients without indications for long-term anticoagulation.

Perspectives **COMPETENCY IN PATIENT CARE:** This study signifies SAPT as the safest and most effective treatment for patients without indication for anticoagulation post-TAVR. It is especially beneficial for older adults with frailty or comorbidities like COPD, where bleeding risks are increased. Careful consideration of individual patient characteristics and risk factors is critical to optimize outcomes.

**TRANSLATIONAL OUTLOOK:** SAPT is associated with less adverse events following TAVR in patients without an indication for anticoagulation. However, the limited number of randomized controlled trials and the observed trend toward higher mortality with DOACs highlight the need for well-powered head-to-head RCTs to further evaluate the role of DOACs and VKAs in this population. These future findings may lead to the refinement of guideline-directed antithrombotic strategies following TAVR.

## Funding support and author disclosures

This research did not receive any specific grant or funding from public, commercial, or not-for-profit sectors. Dr Nanna has received current research support from the American College of Cardiology Foundation, the Patient-Centered Outcomes Research Institute (PCORI), the Yale Claude D. Pepper Older Americans Independence Center (P30AG021342), and the National Institute on Aging (K76AG088428); and is a consultant for Novo Nordisk, Merck, and HeartFlow, Inc. Dr Kolte has received research funding from the NIH/NHLBI, United States and Medtronic, Inc, United States outside of the submitted work. All other authors have reported that they have no relationships relevant to the contents of this paper to disclose.
