# Supplementary material for: Post-Transcatheter Aortic Valve Replacement Antithrombotic Treatment in Nonindicated Patients: Updated Systematic Review and Network Meta-Analysis
Source: JACC Adv. 2025 Mar 31;4(5):101719. doi: 10.1016/j.jacadv.2025.101719 (PMC12049831; doi:10.1016/j.jacadv.2025.101719)
Supplement: Supplementary data [file mmc1.pdf]

Post-Transcatheter Aortic Valve Replacement Antithrombotic Treatment in Non-indicated Patients: Updated Systematic Review and Network Meta-analysis

Supplemental Material

Supplemental Figures

Supplemental Figure 1. Detailed pairwise network meta-analysis of safety and efficacy outcomes across different antithrombotic regimens **2**

Supplemental Figure 2. Meta-regression analysis of the effects of baseline characteristics on all-cause mortality and major or life-threatening bleeding with the comparison-adjusted bubble plots **3**

Supplemental Figure 3. Subgroup analysis of low-dose Rivaroxaban combined with 3-month SAPT **6**

Supplemental Figure 4. Subgroup analysis of DOAC with or without SAPT **7**

Supplemental Figure 5. Sensitivity analysis of RCTs for all-cause mortality, major or life-threatening bleeding, and total bleeding **8**

Supplemental Figure 6. Sensitivity analysis of RCTs for cardiovascular mortality, stroke, and myocardial infarction **9**

Supplemental Figure 7. Forest plot of bayesian network meta-analysis results for all evaluated outcomes **10**

Supplemental Figure 8. Node-splitting analysis of bayesian network meta-analysis for safety and efficacy outcomes across different antithrombotic regimens **11**

Supplemental Figure 9. Funnel plots for assessing publication bias across all outcomes **12**

Supplemental Figure 10. Risk of bias assessment of RCTs using the RoB-2 quality assessment tool **13**

Supplemental Figure 11. Risk of bias assessment of PSM cohorts using the ROBINS-I quality assessment tool **14**

Supplemental Tables

Supplemental Table 1. Search strategies used for PubMed, Embase, and Scopus databases **15**

Supplemental Table 2. Baseline characteristics of participants in included studies **18**

Supplemental Table 3. Eligibility criteria and number of events for each outcome in included studies **20**

Supplemental Table 4. Publication bias assessment using Egger’s and Begg’s tests **22**

Supplemental Table 5. PRISMA 2020 checklist items **23**

Supplemental Figure 1. Detailed pairwise network meta-analysis of safety and efficacy outcomes across different antithrombotic regimens, presenting direct, indirect, and network estimates

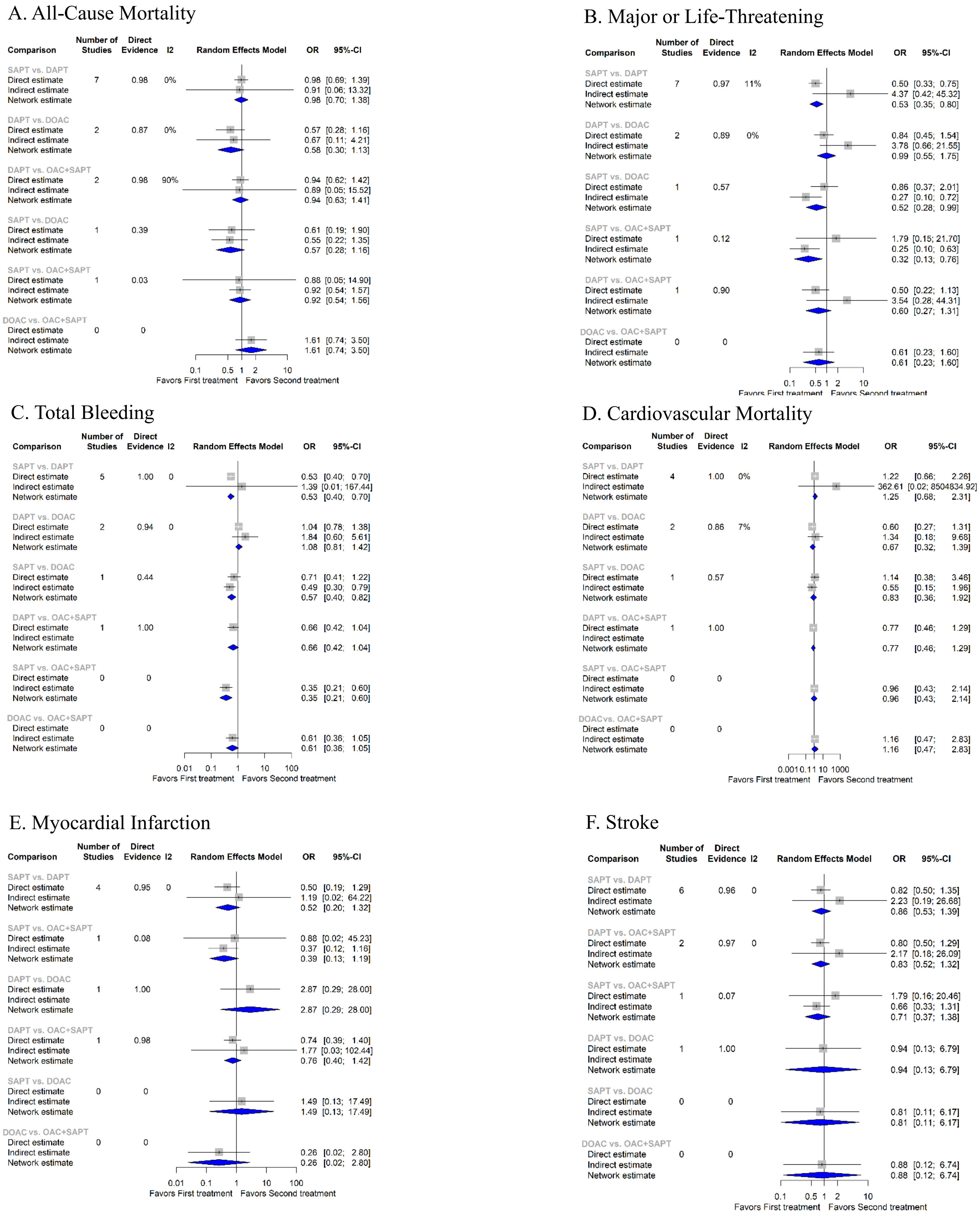

Abbreviations: CI: confidence interval, DAPT: dual antiplatelet therapy, DOAC: direct oral anticoagulant, OAC: oral anticoagulant, OR: odds ratio, SAPT: single antiplatelet therapy.

Supplemental Figure 2. Meta-regression analysis of the effects of baseline characteristics on all-cause mortality (A) and major or life-threatening bleeding (B) with the comparison-adjusted bubble plots

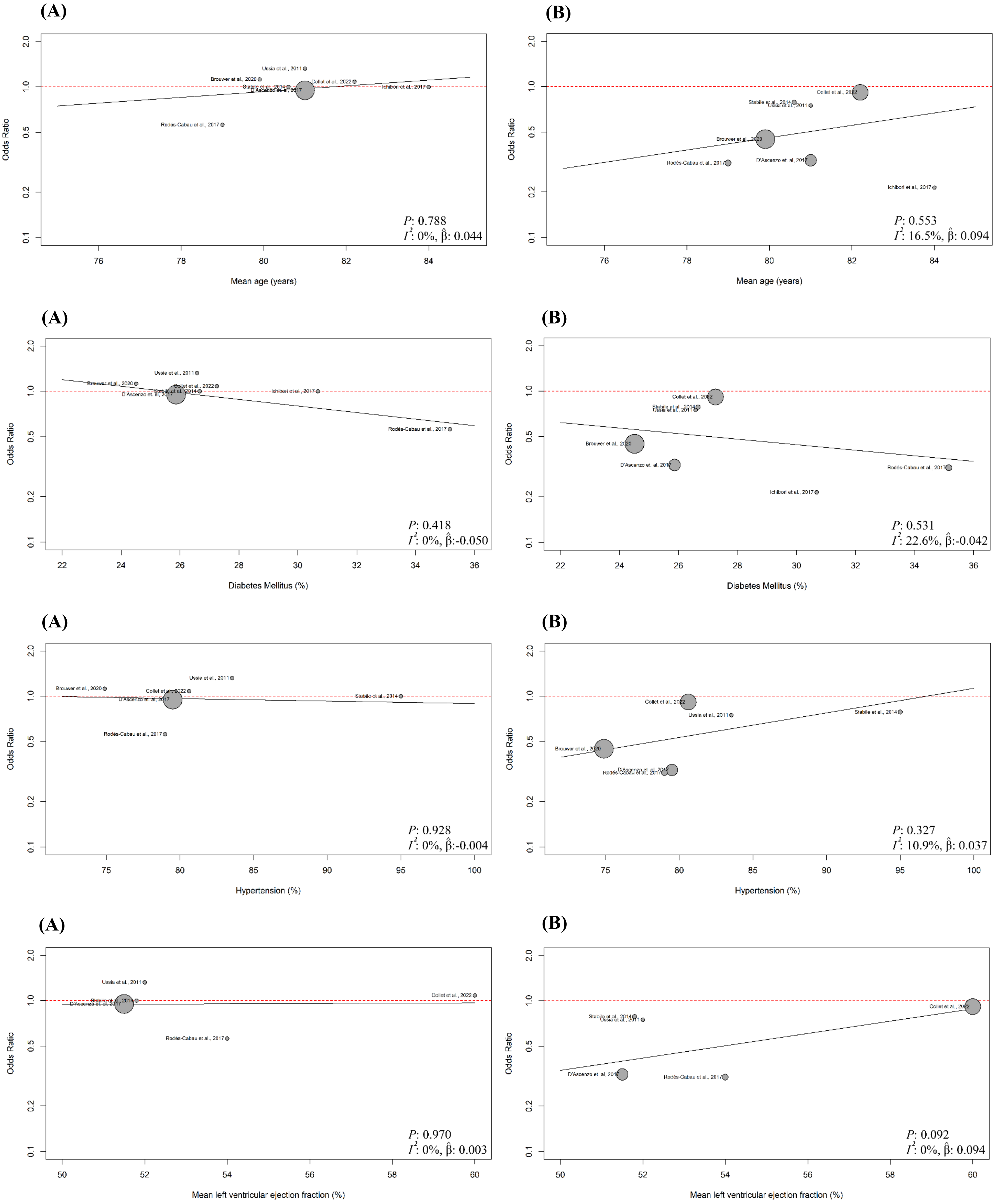

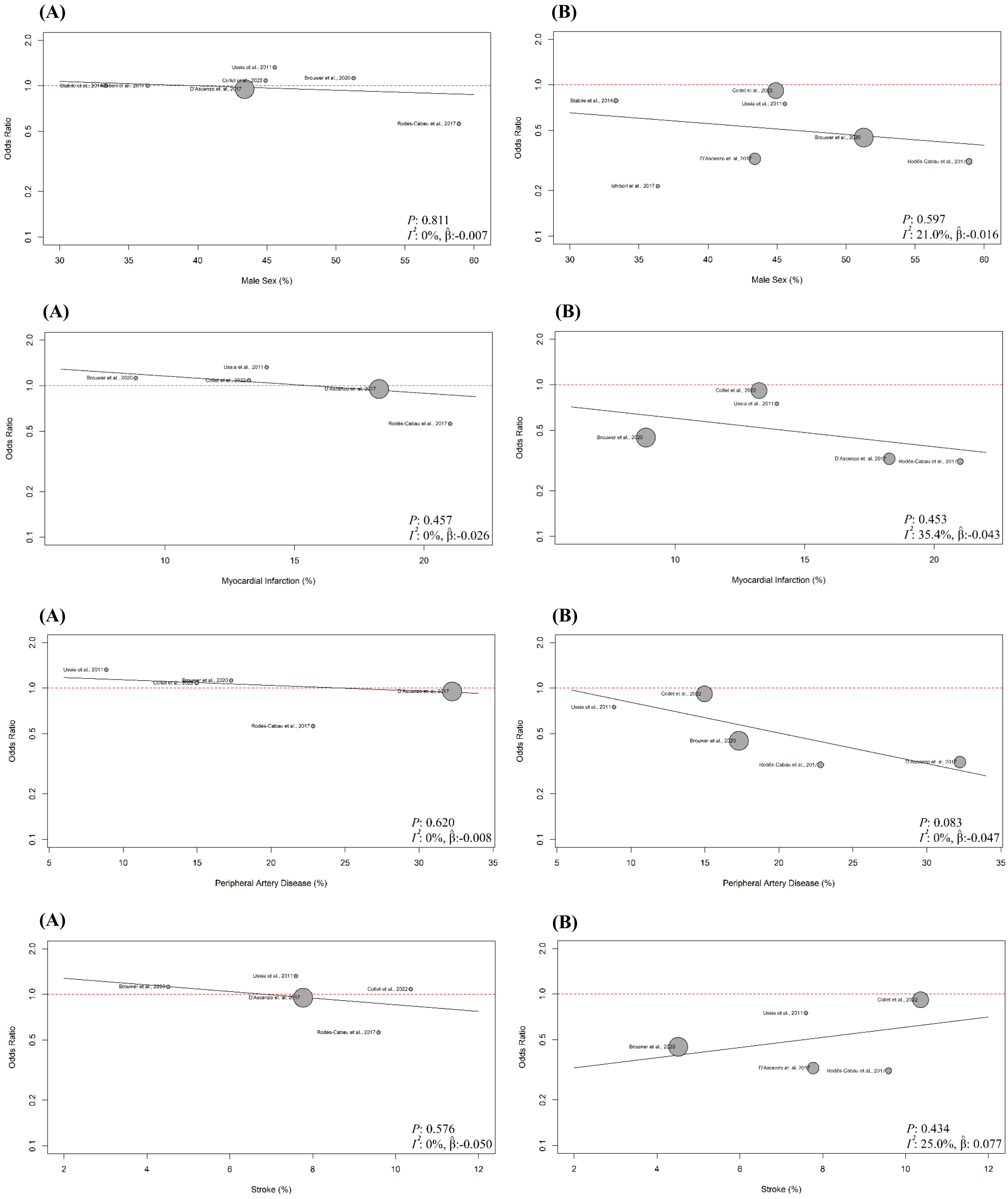
(A)
Odds Ratio
Male Sex (%)
P: 0.811
I2: 0%, β̂:-0.007
(B)
Odds Ratio
Male Sex (%)
P: 0.597
I2: 21.0%, β̂:-0.016
(A)
Odds Ratio
Myocardial Infarction (%)
P: 0.457
I2: 0%, β̂:-0.026
(B)
Odds Ratio
Myocardial Infarction (%)
P: 0.453
I2: 35.4%, β̂:-0.043
(A)
Odds Ratio
Peripheral Artery Disease (%)
P: 0.620
I2: 0%, β̂:-0.008
(B)
Odds Ratio
Peripheral Artery Disease (%)
P: 0.083
I2: 0%, β̂:-0.047
(A)
Odds Ratio
Stroke (%)
P: 0.576
I2: 0%, β̂:-0.050
(B)
Odds Ratio
Stroke (%)
P: 0.434
I2: 25.0%, β̂: 0.077

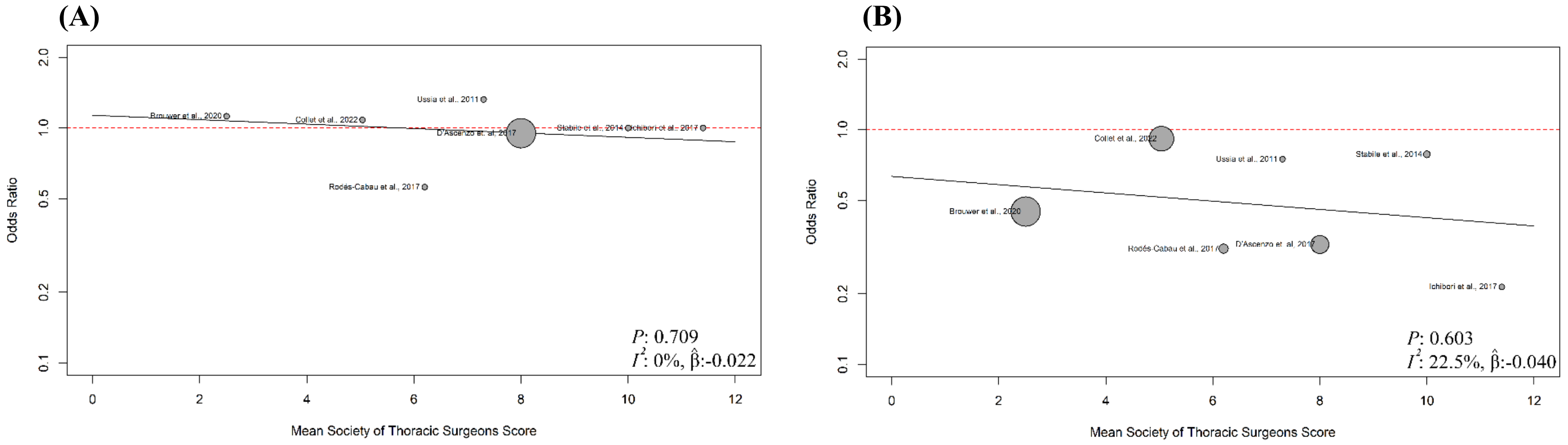
(A)
Odds Ratio
Mean Society of Thoracic Surgeons Score
Brouwer et al., 2020
Collet et al., 2022
Ussia et al., 2011
D'Ascenzo et al., 2017
Stabile et al., 2014
Ichibori et al., 2017
Rodés-Cabau et al., 2017
P: 0.709
I²: 0%, β̂:-0.022
(B)
Odds Ratio
Mean Society of Thoracic Surgeons Score
Collet et al., 2022
Ussia et al., 2011
Stabile et al., 2014
Brouwer et al., 2020
Rodés-Cabau et al., 2017
D'Ascenzo et al., 2017
Ichibori et al., 2017
P: 0.603
I²: 22.5%, β̂:-0.040

Supplemental Figure 3. Subgroup analysis of low-dose Rivaroxaban combined with 3-month SAPT, separated from other regimens, for all evaluated outcomes

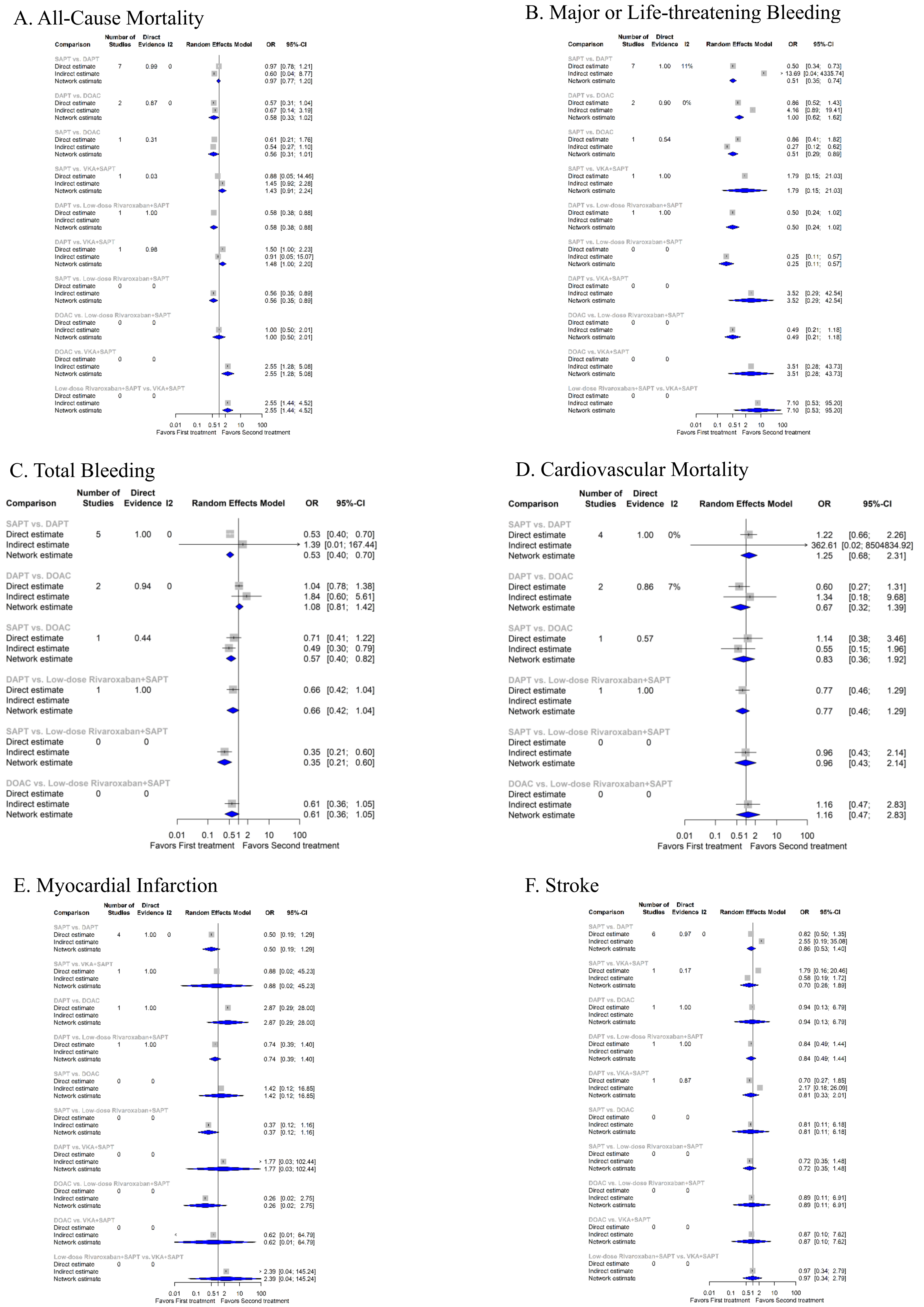

Abbreviations: CI: confidence interval, DAPT: dual antiplatelet therapy, DOAC: direct oral anticoagulant, OAC: oral anticoagulant, OR: odds ratio, SAPT: single antiplatelet therapy.

Supplemental Figure 4. Subgroup analysis of DOAC with or without SAPT, compared with other regimens, for all evaluated outcomes

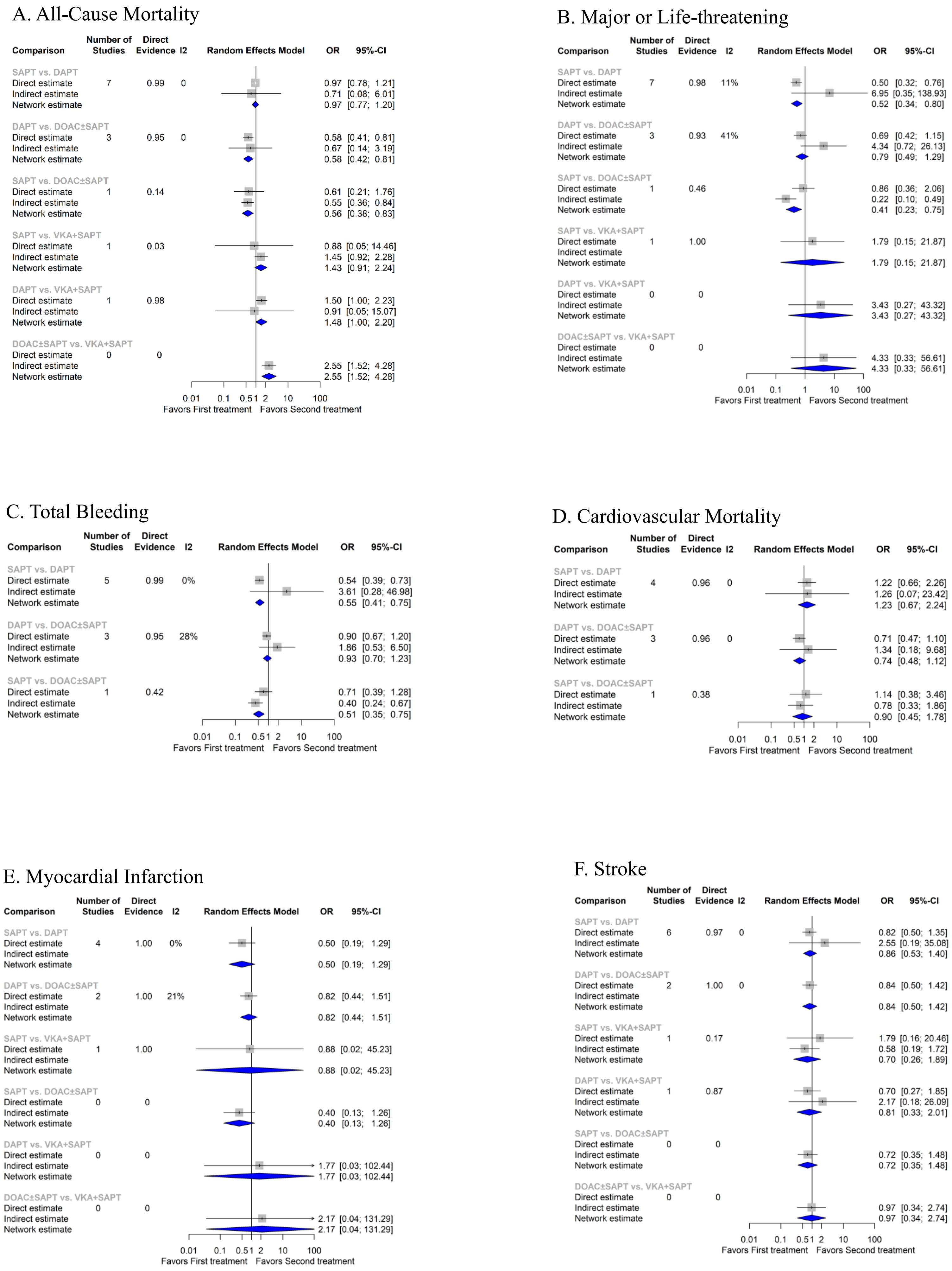

Abbreviations: CI: confidence interval, DAPT: dual antiplatelet therapy, DOAC: direct oral anticoagulant, OAC: oral anticoagulant, OR: odds ratio, SAPT: single antiplatelet therapy.

Supplemental Figure 5. Sensitivity analysis of randomized controlled trials, excluding propensity score-matched cohorts, for all-cause mortality, major or life-threatening bleeding, and total bleeding

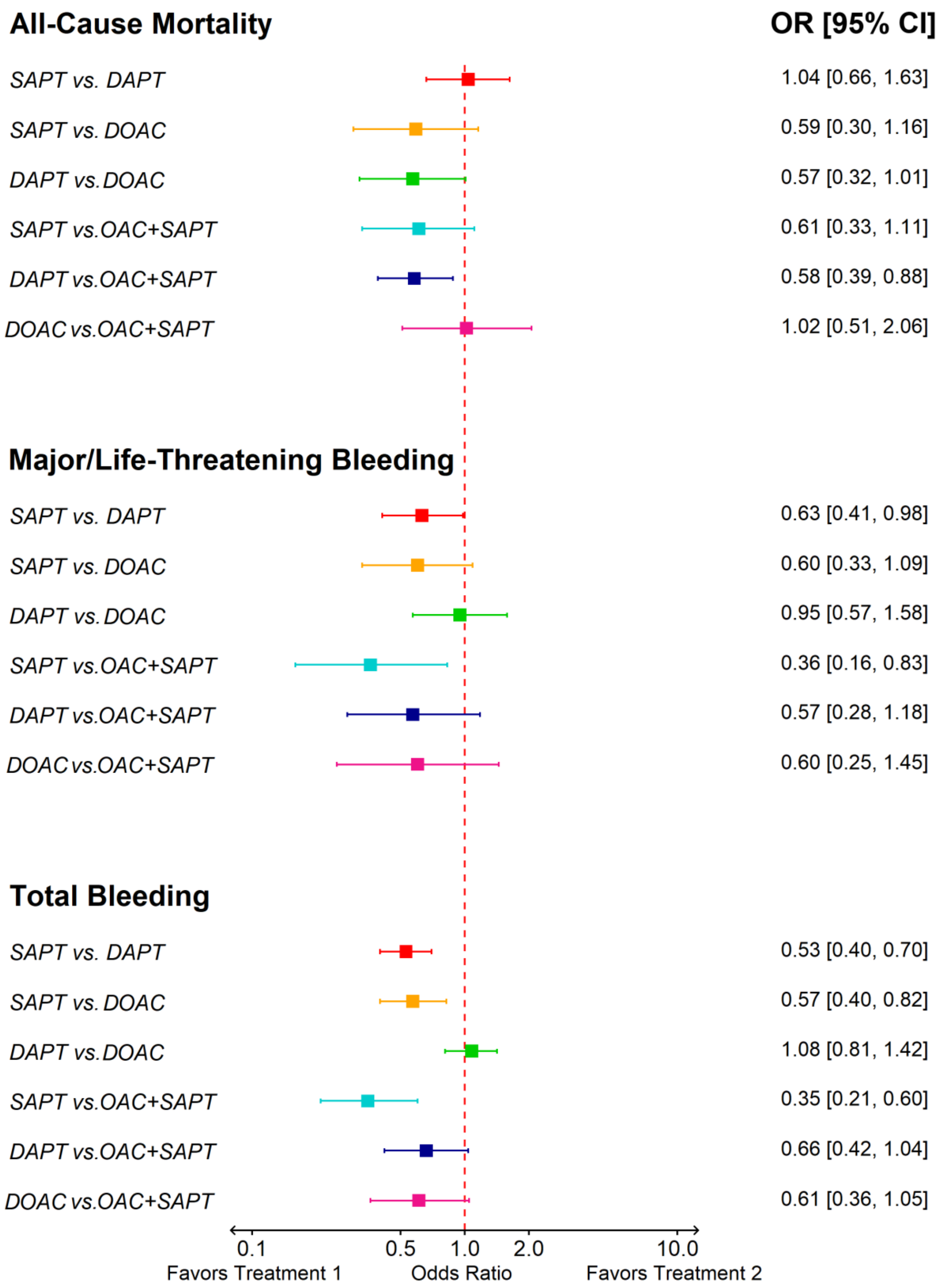

Abbreviations: CI: confidence interval, DAPT: dual antiplatelet therapy, DOAC: direct oral anticoagulant, OAC: oral anticoagulant, OR: odds ratio, SAPT: single antiplatelet therapy.

Supplemental Figure 6. Sensitivity analysis of randomized controlled trials, excluding propensity score-matched cohorts, for cardiovascular mortality, stroke, and myocardial infarction

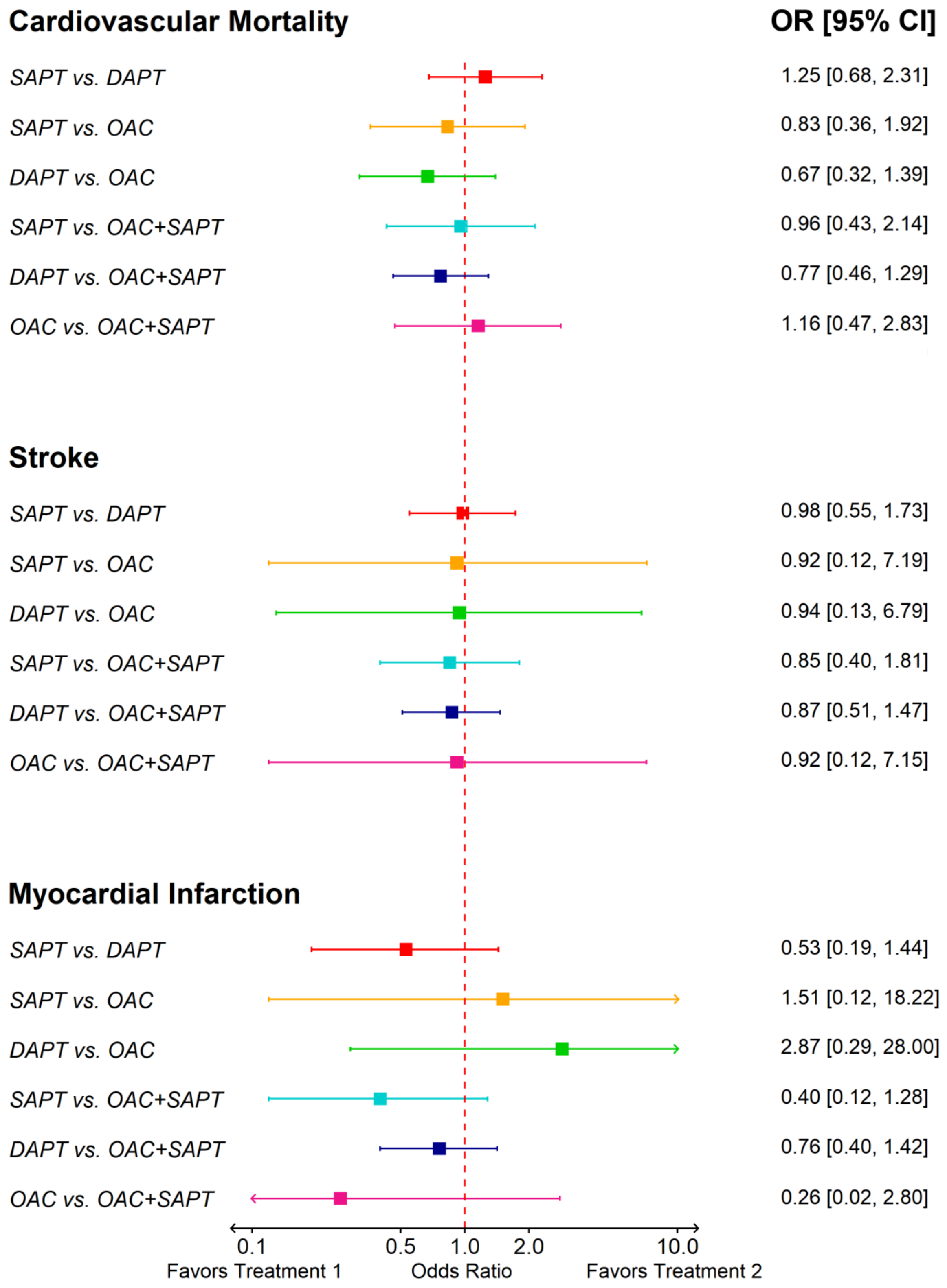

Abbreviations: CI: confidence interval, DAPT: dual antiplatelet therapy, DOAC: direct oral anticoagulant, OAC: oral anticoagulant, OR: odds ratio, SAPT: single antiplatelet therapy.

Supplemental Figure 7. Forest plot of bayesian network meta-analysis results for all evaluated outcomes, comparing treatments to SAPT (reference)

A. All-Cause Mortality
(Potential scale reduction factor: 1.00028)

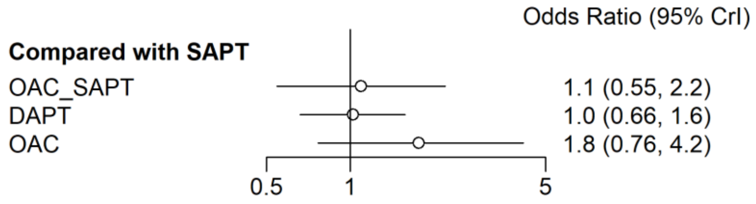

B. Major or Life-threatening Bleeding
(Potential scale reduction factor: 1.00046)

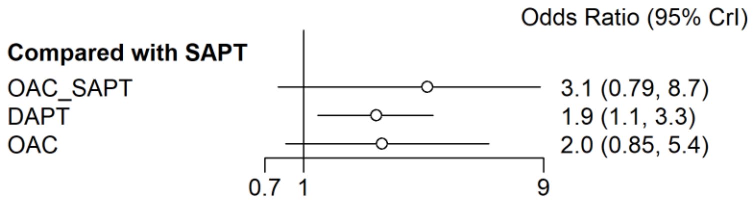

C. Total Bleeding
(Potential scale reduction factor: 1.00010)

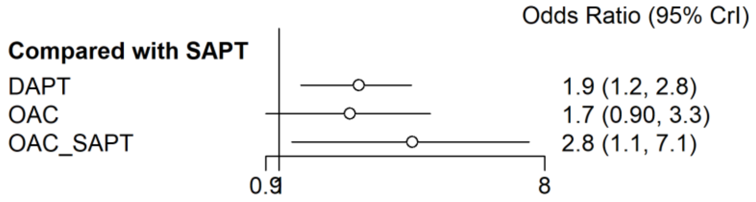

D. Cardiovascular Mortality
(Potential scale reduction factor: 1.00041)

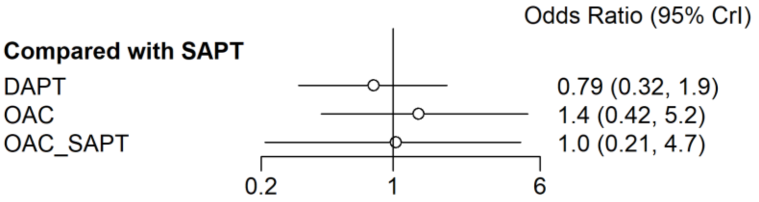

E. Myocardial Infarction
(Potential scale reduction factor: 1.00084)

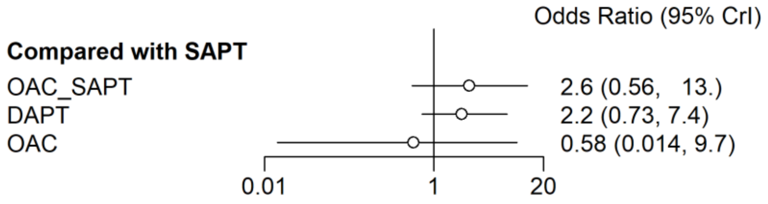

F. Stroke
(Potential scale reduction factor: 1.00115)

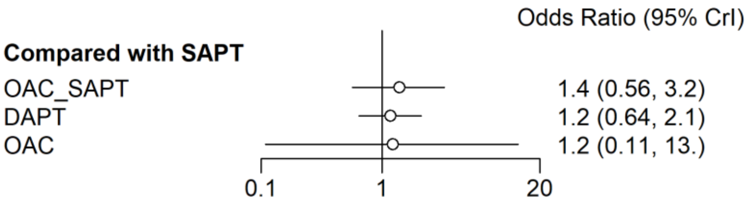

Points represent posterior mean odds ratios, with 95% credible intervals shown as horizontal lines.

Abbreviations: CI: credible interval, DAPT: dual antiplatelet therapy, OAC: oral anticoagulant, OR: odds ratio, SAPT: single antiplatelet therapy

Supplemental Figure 8. Node-splitting analysis of bayesian network meta-analysis for safety and efficacy outcomes across different antithrombotic regimens, presenting direct, indirect, and network estimates

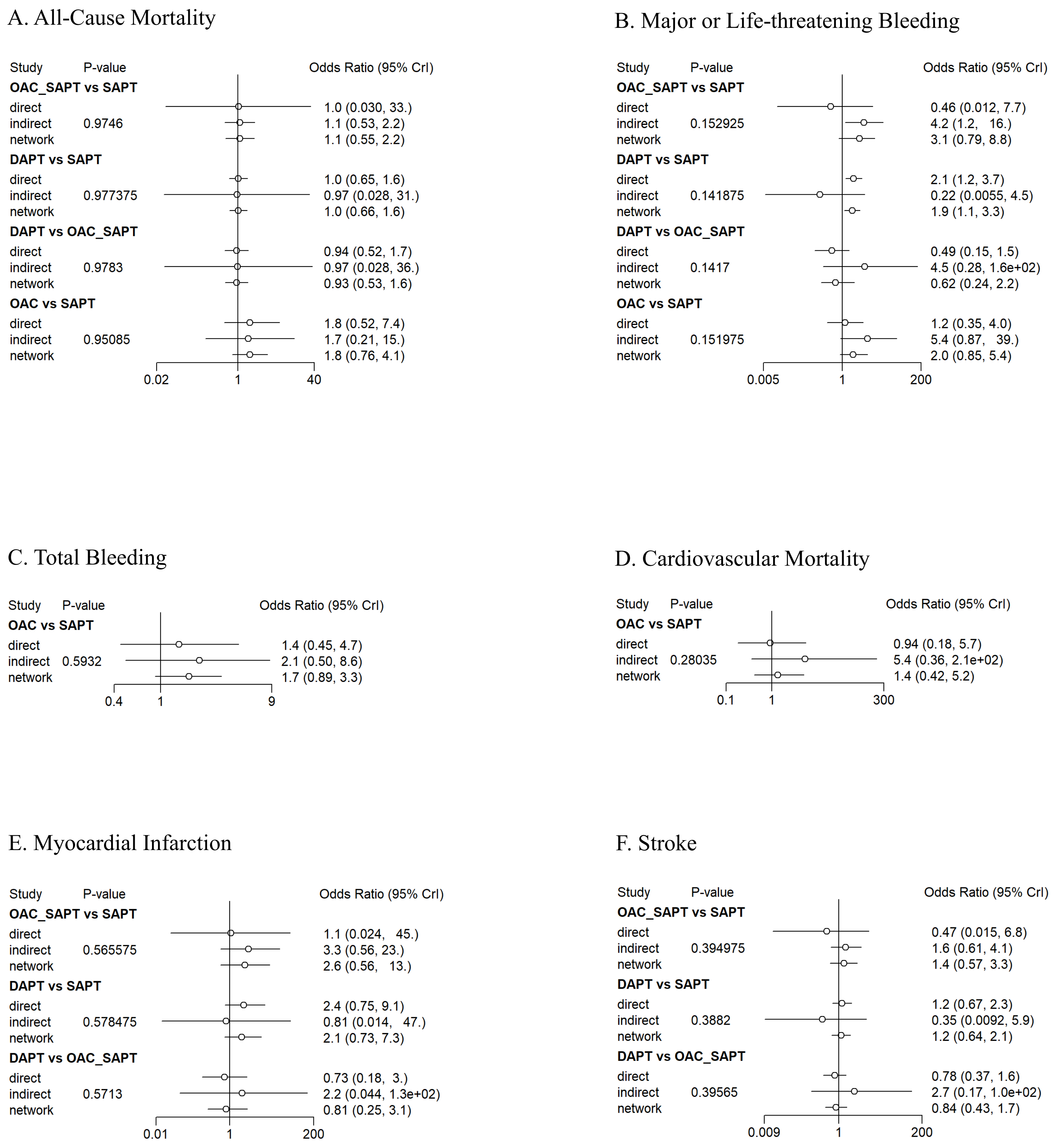

P-value >0.05 indicates consistency, suggesting agreement between direct and indirect evidence.

Abbreviations: CI: confidence interval, DAPT: dual antiplatelet therapy, DOAC: direct oral anticoagulant, OAC: oral anticoagulant, OR: odds ratio, SAPT: single antiplatelet therapy.

Supplemental Figure 9. Funnel plots for assessing publication bias across all outcomes

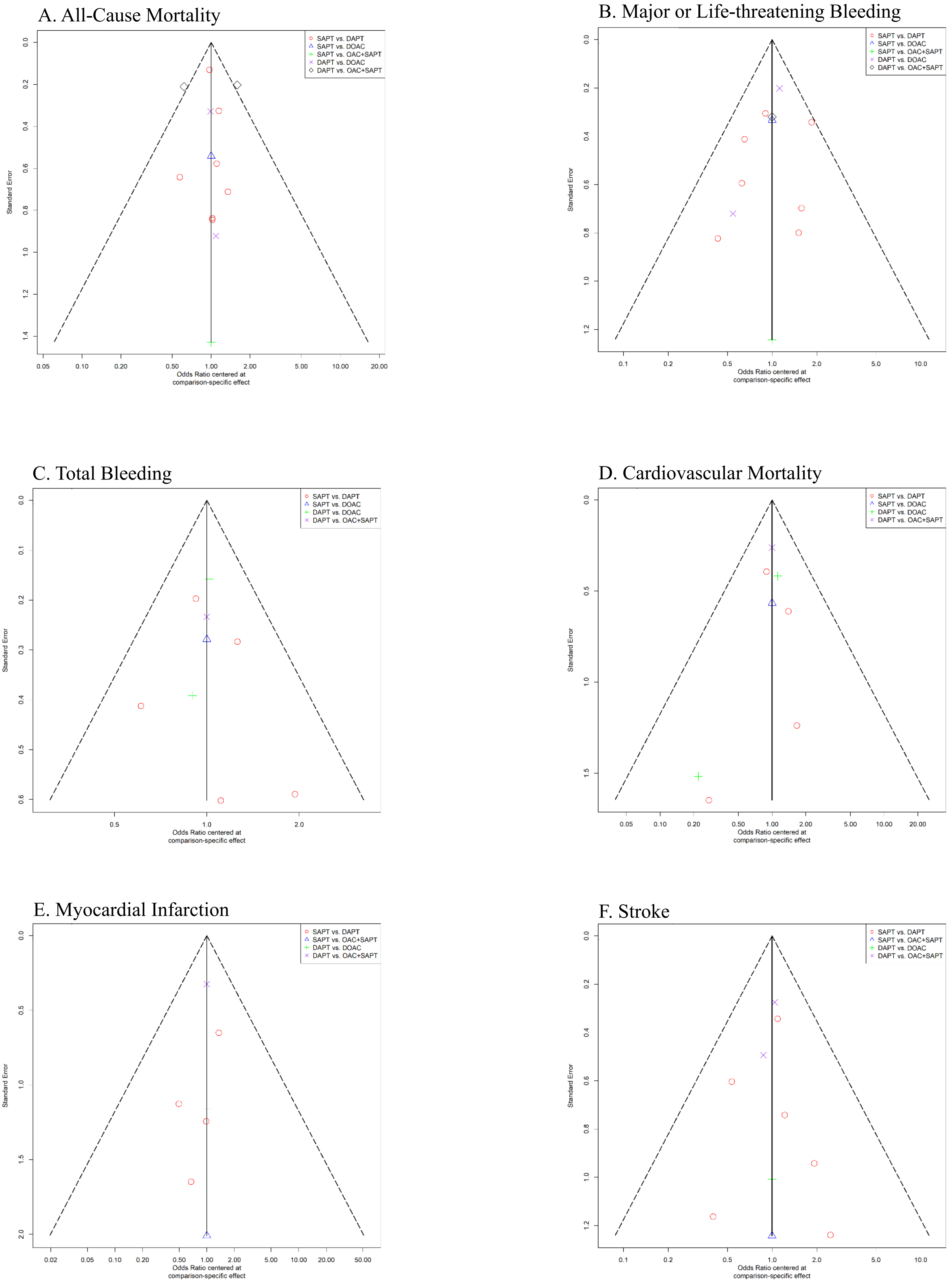

Abbreviations: CI: confidence interval, DAPT: dual antiplatelet therapy, DOAC: direct oral anticoagulant, OAC: oral anticoagulant, OR: odds ratio, SAPT: single antiplatelet therapy.

Supplemental Figure 10. Risk of bias assessment of randomized controlled trials using the RoB-2 quality assessment tool. (A) Risk of bias evaluation for included trials. (B) Proportion of trials classified as low risk, high risk, or having some concerns for each RoB-2 evaluation criterion

(A)

Study	D1	D2	D3	D4	D5	Overall
Ussia, 2011	!	+	+	+	+	!
Stabile, 2014	!	+	+	+	+	!
Rodés-Cabau, 2017	+	+	+	+	+	+
Brouwer, 2020	+	!	+	+	+	!
Dangas, 2020	!	!	+	+	-	-
Rogers, 2021	+	+	+	+	!	!
Collet, 2022	+	+	!	+	+	!
Park, 2022	+	+	+	+	+	+
Merdler, 2023	+	-	+	+	!	-

+ Low risk
! Some concerns
- High risk

D1 Randomisation process
D2 Deviations from the intended interventions
D3 Missing outcome data
D4 Measurement of the outcome
D5 Selection of the reported result

(B)

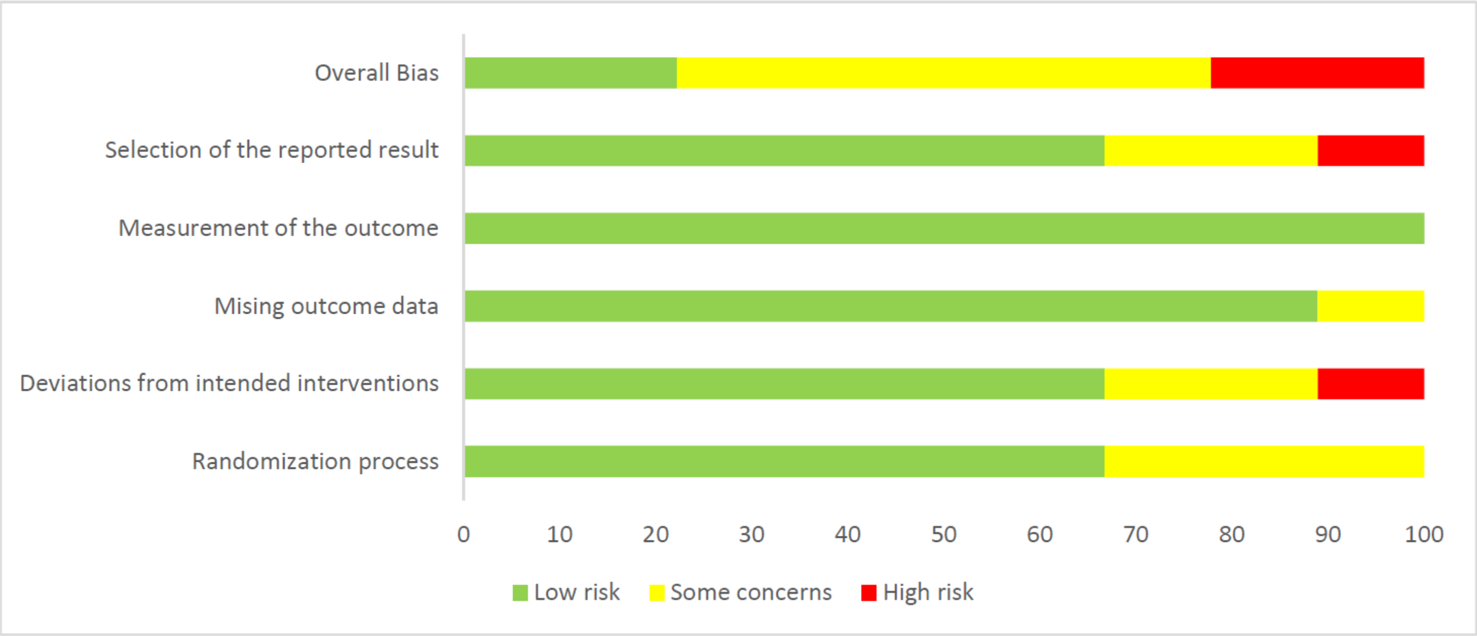

Supplemental Figure 11. Risk of bias assessment of propensity score-matched cohorts using the ROBINS-I quality assessment tool

Study	Bias due to confounding	Bias in selection of participants into the study	Bias in classification of interventions	Bias due to deviations from intended intervention	Bias due to missing data	Bias in measurement of outcomes	Bias in selection of the reported result	overall
D'Ascenzo 2017	Moderate	Low	Moderate	Low	Low	Moderate	Low	Moderate
Ichibori 2017	Moderate	Moderate	Low	Low	Low	Low	Low	Moderate
Naser 2023	Moderate	Moderate	Low	Low	Low	Moderate	Moderate	Moderate

Supplemental Table 1. Search strategies used for PubMed, Embase, and Scopus databases

PubMed (August 2024)		
#1	TAVR[tiab] OR “Transcatheter Aortic Valve Implantat*”[tiab] OR TAVR[tiab] OR “Transcatheter Aortic Valve Replacement”[tiab] OR "Transcatheter Aortic Valve Replacement"[MeSH Terms] OR "valve-in-valve transcatheter aortic valve implant*"[tiab] OR "valve-in valve trans-catheter aortic valve*"[tiab] OR "valve-in-valve transcatheter aortic valve*"[tiab] OR "transcatheter aortic valve-in-valve implant*"[tiab] OR "valve in valve TAV*"[tiab] OR "aortic valve implant*"[tiab] OR “trans-catheter aortic valve*"[tiab] OR “transcatheter aortic valve*"[tiab]	
#2	"Antithrombins"[Mesh] OR Antithrombin*[Title/Abstract] OR antithrombotic*[tiab] OR “Direct Thrombin Inhibitor*”[tiab] OR “antiplatelet therapy”[tiab] OR "antiplatelet*"[tiab] OR "Platelet Aggregation Inhibitors"[Mesh] OR "platelet aggregation inhibit*"[tiab] OR "platelet inhibitor*"[tiab] OR “Blood Platelet Aggregation Inhibitor*”[Title/Abstract] OR “Platelet Antiaggregant*”[Title/Abstract] OR “Platelet Antagonist*”[Title/Abstract] OR “Antiplatelet Agent*”[Title/Abstract] OR “Antiplatelet Drug*”[Title/Abstract] OR “Protease-Activated Receptor-1 Antagonist*”[Title/Abstract] OR “PAR-1 Antagonist*”[Title/Abstract] OR DAPT[tiab] OR "Dual Anti-Platelet Therapy"[Mesh] OR "Dual Antiplatelet Therap*"[tiab] OR "Dual Anti-Platelet Therap*”[tiab] OR "Dual Anti Platelet Therap*"[tiab] OR “Dual Antiplatelet Treat*”[tiab] OR "Drug Therapy, Combination"[Mesh] OR “Combination Drug Therapy”[tiab] OR "combination antiplatelet therapy"[tiab] OR “Drug Therapy, Combination”[Title/Abstract] OR SAPT[tiab] OR "Single Antiplatelet Therap*"[tiab] OR "Single Anti-platelet Therap*"[tiab] OR “Monotherapy”[tiab] OR “Single-agent antiplatelet*”[tiab] OR “Single antithrombotic therap*”[tiab] OR “Single antiplatelet regimen*”[tiab] OR "aspirin"[MeSH Terms] OR aspirin[tiab] OR “Acetylsalicylic Acid”[tiab] OR Solprin[Title/Abstract] OR Polopirin[Title/Abstract] OR Solupsan[Title/Abstract] OR Easprin[Title/Abstract] OR Zorprin[Title/Abstract] OR Acetysal[Title/Abstract] OR Acylpyrin[Title/Abstract] OR Colfarit[Title/Abstract] OR Ecotrin[Title/Abstract] OR Endosprin[Title/Abstract] OR Magnecyl[Title/Abstract] OR Micristin[Title/Abstract] OR Polopiryna[Title/Abstract] OR "Purinergic P2Y Receptor Antagonists"[Mesh] OR "p2y12 inhibitor*"[tiab] OR "p2y12 receptor inhibitor*"[tiab] OR "p2y12 receptor antagonist*"[tiab] OR "Clopidogrel"[Mesh] OR Clopidogrel [tiab] OR Plavix[tiab] OR “Clopidogrel Bisulfate”[Title/Abstract] OR Iscover[Title/Abstract] OR “Clopidogrel Napadisilate”[Title/Abstract] OR “Clopidogrel Besylate”[Title/Abstract] OR “Clopidogrel Hydrochloride”[Title/Abstract] OR “Clopidogrel Sandoz”[Title/Abstract] OR “Clopidogrel Mepha”[Title/Abstract] OR "Ticagrelor"[Mesh] OR Ticagrelor[tiab] OR Brilique[Title/Abstract] OR Brilinta[Title/Abstract] OR "Prasugrel Hydrochloride"[Mesh] OR Prasugrel[tiab] OR “Hydrochloride, Prasugrel”[Title/Abstract] OR “Prasugrel HCl”[Title/Abstract] OR Ticlopidine[MeSH Terms]	
#3	"anticoagulants"[MeSH Terms] OR Anticoagulant*[tiab] OR “Anticoagulant Therapy”[tiab] OR anticoagul*[tiab] OR “Indirect Thrombin Inhibitor*”[tiab] OR OAC[tiab] OR “Oral Anticoagul*”[tiab] OR DOAC*[tiab] OR “Direct Oral Anticoagulant*”[tiab] OR "Factor Xa Inhibitors"[Mesh] OR “Factor Xa Inhibitor*”[tiab] OR "direct acting oral anticoagulant*"[tiab] OR "Direct Factor Xa Inhibitor*"[tiab] OR NOAC*[tiab] OR “Novel Oral Anticoagulant*”[tiab] OR “Non-vitamin K Oral Anticoagulant*”[tiab] OR "New Oral Anticoagulant*"[tiab] OR "dabigatran"[MeSH Terms] OR dabigatran[tiab] OR “Dabigatran Etexilate”[Title/Abstract] OR Pradaxa[Title/Abstract] OR “Dabigatran Etexilate Mesylate”[Title/Abstract] OR "apixaban"[Supplementary Concept] OR apixaban[tiab] OR "rivaroxaban"[MeSH Terms] OR rivaroxaban[tiab] OR Xarelto[Title/Abstract] OR "edoxaban"[Supplementary Concept]OR edoxaban[tiab] OR Savaysa[tiab] OR VKA[tiab] OR “vitamin k antagonist*”[tiab] OR "Warfarin"[Mesh] OR Warfarin[tiab] OR “Warfarin Potassium”[Title/Abstract] OR “Warfarin Sodium”[Title/Abstract] OR Coumadin[Title/Abstract] OR Marevan[Title/Abstract] OR Aldocumar[Title/Abstract] OR Coumadine[Title/Abstract] OR “Apo-Warfarin”[Title/Abstract]	
#4	#1 AND (#2 OR #3)	963

Scopus (August 2024)		
#1	"transcatheter aortic valve implant*" OR "transcatheter aortic valve replacement" OR "percutaneous aortic valve implant*" OR "percutaneous aortic valve replacement" OR TAVR OR TAVR OR "trans-apical aortic valve implant*" OR "trans-apical aortic valve replacement" OR "trans-arterial aortic valve implant*" OR "trans-arterial aortic valve replacement" OR "trans-catheter aortic valve implant*" OR "trans-catheter aortic valve replacement" OR "trans-cutaneous aortic valve implant*" OR "trans-cutaneous aortic valve replacement" OR "trans-femoral aortic valve implant*" OR "trans-femoral aortic valve replacement" OR "transapical aortic valve implant*" OR "transapical aortic valve replacement" OR "transarterial aortic valve implant*" OR "transarterial aortic valve replacement" OR "transcatheter aortic valve replacement" OR "transcutaneous aortic valve implant*" OR "transcutaneous aortic valve replacement" OR "transfemoral aortic valve implant*" OR "transfemoral aortic valve replacement" OR "valve-in-valve transcatheter aortic valve implant*" OR "percutaneous valve-in-valve implantation of TAVR" OR "TAVR valve-in-valve" OR "TAVR-VIV" OR "TAVR-VIV" OR "transcatheter aortic valve replacement (TAVR)" OR "transcatheter aortic valve replacement (TAVR) valve-in-valve (V-i-V)" OR "transcatheter aortic valve replacement (TAVR) valve-in-valve (ViV) therapy" OR "transcatheter aortic valve replacement valve-in-valve procedure" OR "transcatheter aortic valve-in-valve (TAVR-ViV) implant*" OR "transcatheter aortic valve-in-valve implant* (TAVR-ViV)" OR "transcatheter aortic valve-in-valve implant* (VinV-TAVR)" OR "transcatheter aortic valve-in-valve implant* (ViV TAVR)" OR "valve in valve (ViV) TAV*" OR "valve in valve TAV*" OR "valve in valve trans-catheter aortic valve replacement" OR "valve-in valve (ViV) trans-catheter aortic valve implant* (TAVR)" OR "valve-in-valve (VinV) TAV*" OR "valve-in-valve (viv) transcatheter aortic implant* (TAVR)" OR "valve-in-valve (viv) transcatheter aortic valve implant* (TAVR)" OR "valve-in-valve (ViV) transcatheter aortic valve replacement (TAVR)" OR "valve-in-valve therapy (ViV-TAVR)" OR "valve-in-valve trans-catheter aortic valve replacement" OR "valve-in-valve transcatheter aortic implant*" OR "valve-in-valve transcatheter aortic valve replacement" OR "valve-in-valve transcatheter aortic valve replacement (ViV-TAVR)" OR "valve-in-valve transcatheter valve implant* (TAVR)" OR "ViV transcatheter aortic valve implant*" OR "ViV-TAV*" OR "VinV-TAV*"	
#2	"antithrombocytic agent*" OR "antiplatelet agent*" OR "antiplatelet drug*" OR "platelet aggregation inhibitor*" OR "platelet inhibit*" OR "thrombocyte aggregation inhibit*" OR "Blood Platelet Aggregation Inhibitor*" OR "Blood Platelet Ant*" OR "Platelet Ant*" OR "single antiplatelet therapy" OR SAPT OR "dual antiplatelet therapy" OR "antiplatelet combination therapy" OR "combination antiplatelet therapy" OR "dual anti-platelet therapy" OR DAPT OR "anticoagula* agent*" OR "anti coagula*" OR "anti coagula* drug*" OR "anti coagula* agent*" OR anticoagula* OR "anticoagula* drug*" OR antithromb* OR "antithromb* agent*" OR "antithromb* drug*" OR "oral anticoagula*" OR "oral anticoagula* agent*" OR "direct oral anticoagula* agent*" OR "direct oral anticoagula*" OR DOAC OR "novel oral anticoagula*" OR "new oral anticoagula*" OR NOAC OR Dabigatran OR Rivaroxaban OR Argatroban OR Edoxaban OR Warfarin OR "warfarin potassium" OR "warfarin sodium" OR "anti vitamin k" OR "menadione antagonist" OR "vitamin K antagonist*" OR "antivitamin* K" OR VKA OR "acetylsalicylic acid" OR asa OR aspirin OR Clopidogrel OR osvix OR plavitor OR plavix OR pregrel OR Ticagrelor OR brilinta OR brilique OR possia OR Prasugrel OR "Prasugrel Hydrochloride" OR Tirofiban OR Eptifibatide OR "purinergic P2Y receptor antagonist*" OR "P2Y purinoceptor antagonist*" OR "p2y12 receptor inhibit*" OR "p2y12 receptor antagonist*" OR "p2y12 inhibit*"	
#3	"major adverse cardi* event*" OR "major cardi* adverse event*" OR "adverse cardi* event*" OR MACE OR MCAE OR "cardi* dis*" OR "cardi* complication*" OR "cardi* event*" OR Stroke* OR "Cerebr* Accident*" OR "Cerebr* Stroke*" OR "Brain Vascular Accident*" OR CVA OR "Acute Stroke*" OR "Acute Cerebr* Accident*" OR "CVA* (Cerebrovascular Accident)" OR "apoplexy" OR "Cerebr* Apoplexy" OR apoplexia OR "cerebr* apoplexia" OR "apoplectic stroke*" OR "acute cerebr* lesion*" OR "thrombotic stroke*" OR "cerebr* vascular accident*" OR "brain accident*" OR "brain attack*" OR "cerebr* injury" OR "heart arrhythmia*" OR "arrhythmia*" OR "arrythmia*" OR "cardiac arr*" OR "heart arr*" OR "heart dysrhythmia*" OR "heart rhythm dis*" OR "cardiac dysrhythmia*" OR "cardiac disrhythmia*" OR "Cardial arrhythmia*" OR "ectopic heart rhythm*" OR "ectopic rhythm*" OR "heart aberrant conduction" OR "heart ectopic beat" OR "heart rhythm problem*" OR "myocardial arrhythmia*" OR "cardia* valve defect" OR "cardia* valv* dis*" OR "heart valve abnorm*" OR "heart valv* defect" OR "heart valv* degeneration" OR "heart valv* dis*" OR "heart valve lesion" OR "Valv* Heart Dis*" OR "valvulopath*" OR "aort* valv* defect" OR "aort* valv* dis*" OR "aorta valve lesion" OR	

	"aortic valv* insufficiency" OR "aortic valvulopath*" OR "Aortic Valvular Heart Dis*" OR "Aortic Heart Dis*" OR HALT OR "hypoattenuated leaflet thickening" OR "Myocardi* Infarct*" OR "Cardiovascular Stroke*" OR "cardia* infarct*" OR "heart infarct*" OR "heart micro infarct*" OR "heart muscle infarct*" OR "Heart Attack" OR "Heart Failure" OR "Cardiac Failure" OR HF OR MI))	
#4	(TITLE-KEY-ABS): #1 AND #2 AND #3	2,212

Embase (August 2024)		
#1	('transcatheter aortic valve implantation'/syn OR 'transcatheter aortic valve implantation':ti,ab,kw OR 'tav*':ti,ab,kw)	
#2	('anticoagula*':ti,ab,kw OR 'anti*coagula*':ti,ab,kw OR 'antithrombo*':ti,ab,kw OR 'anti*thrombo*':ti,ab,kw OR 'antiplatelet*':ti,ab,kw OR 'anti*platelet*':ti,ab,kw OR 'anticoagulant agent'/syn OR 'anticoagulant agent':ti,ab,kw OR 'anticoagulation'/syn OR 'anticoagulation':ti,ab,kw OR 'anticoagulant therapy'/syn OR 'anticoagulant therapy':ti,ab,kw OR 'antithrombocytic agent'/syn OR 'antithrombocytic agent':ti,ab,kw OR 'dual antiplatelet therapy'/syn OR 'dual antiplatelet therapy':ti,ab,kw OR 'antiplatelet therapy'/syn OR 'antiplatelet therapy':ti,ab,kw OR 'single antiplatelet therapy'/syn OR 'acetylsalicylic acid'/syn OR 'clopidogrel'/syn OR 'blood clotting inhibitor'/syn OR 'antivitamin k'/syn OR 'warfarin'/syn OR 'ticagrelor'/syn OR 'prasugrel'/syn)	
#3	('major adverse cardiac event'/syn OR 'major cardiac adverse event*':ti,ab,kw OR 'mace':ti,ab,kw OR 'mace' OR 'stroke*':ti,ab,kw OR 'cva*':ti,ab,kw OR 'cerebr*vascular*':ti,ab,kw OR 'cerebrovascular accident'/syn OR 'hypoattenuated leaflet thickening'/syn OR 'halt':ti,ab,kw)	
#4	#1 AND #2 AND #3	1709

Total number of studies	***4,884***
Total number of studies without duplicates	***3,122***

Supplemental Table 2. Baseline characteristics of participants in included studies

Study	Age (years) Mean ± SD		Sex (Female or Male) N (%)		BMI Mean ± SD		STS Score Mean ± SD or Median [IQR]		Comorbidities N (%)												Implanted Valve Type
									DM		HTN		CHF		Stroke/TIA		Prior MI		Previous PCI		
	Arm 1	Arm 2	Arm 1	Arm 2	Arm 1	Arm 2	Arm 1	Arm 2	Arm 1	Arm 2	Arm 1	Arm 2	Arm 1	Arm 2	Arm 1	Arm 2	Arm 1	Arm 2	Arm 1	Arm 2	
**SAPT vs. DAPT**																					
Ussia et al., 2011	81±4	80±6	Female 23 (59)	Female 20 (50)	N/A	N/A	7.3±4	8±5	8 (21)	13 (33)	31 (80)	35 (88)	14 (36)	18 (45)	6 (15.3)	4 (10)	4 (10)	7 (18)	9 (23)	12 (30)	CoreValve Revalving system (SEV)
Stabile et al., 2014 [SAT-TAVR]	81.1±4.8	80.2±5.7	Female 36 (60)	Female 44 (66.7)	N/A	N/A	10.4±6.8	9.7±5.1	17 (28.3)	15 (25.0)	57 (95.0)	57 (95.0)	N/A	N/A	N/A	N/A	N/A	N/A	14 (23.3)	13 (21.3)	SAPIEN XT-Novaflex Delivery System (BEV)
D'Ascenzo et al, 2017	81±4	81±5	Male 256 (37)	Male 269 (38)	26±6	25±7	8±6	8±7	154 (26)	159 (26)	495 (82)	467 (77)	N/A	N/A	N/A	N/A	103	118	N/A	N/A	Edwards SAPIEN and SAPIEN XT valve (BEV)
Ichibori et al., 2017	84±6	84±5	Male 14	Male 18	N/A	N/A	11.6±9.2	11.2±9.0	13	14	N/A	N/A	N/A	N/A	N/A	N/A	N/A	N/A	11	12	SAPIEN or SAPIEN XT heart valve (BEV)
Rodés-Cabau et al., 2017 [ARTE]	79±9	79±9	Male 59 (53.2)	Male 70 (63.1)	N/A	N/A	6.4±4.6	6.2±4.4	36 (32.7)	41 (36.9)	87 (79.8)	86 (77.5)	N/A	N/A	N/A	N/A	20 (18.4)	26 (23.4)	N/A	N/A	Edwards SAPIEN XT or SAPIEN 3 valve (BEV)
Brouwer et al., 2020 [Cohort A POPular TAVR]	80.4±6.2	79.5±6.4	Female 164 (49.5)	Female 160 (47.9)	27.0±4.7	27.1±4.6	2.6 [1.6–3.7]	2.4 [1.7–3.7]	78 (23.6)	85 (25.4)	243 (73.4)	255 (76.3)	N/A	N/A	18 (5.4)	12 (3.6)	28 (8.5)	31 (9.3)	N/A	N/A	Not specified
**SAPT vs. OAC+SAPT**																					
Merdler et al. 2023 [LRT 2.0]*	73.1±5.7	73.6±4.0	Male 37 (74)	Male 29 (65.9)	30.1±5.6	32.0±6.8	1.5±0.5	1.4±0.5	15 (30)	17 (38.6)	39 (78)	36 (81.8)	N/A	N/A	0 (0)	1 (2.3)	2 (4)	0 (0)	6 (12)	4 (9.1)	SEV/BEV
**DAPT vs. OAC**																					
Park et al., 2022 [ADAPT-TAVR]	80±5.3	80.2±5.2	Male 47 (39.8)	Male 49 (44.1)	24.8±4.3	24.8±3.8	3.5±2.7	3.1±2.1	36 (30.5)	35 (31.5)	84 (71.2)	81 (73.0)	12 (10.2)	17 (15.3)	N/A	N/A	2 (1.7)	1 (0.9)	14 (11.9)	18 (16.2)	SAPIEN 3/ Evolut R/ CoreValve/ Evolut PRO/ Acurate Neo (SEV/BEV)
**DAPT vs. OAC+SAPT**																					
Dangas et al., 2020 [GALILEO]	80.8±6.0	80.4±7.1	Male 405 (49.5)	Male 426 (51.6)	28.2±5.7	28.1±5.5	4.3±3.5	4.0±3.2	235 (28.7)	236 (28.6)	697 (85.2)	720 (87.2)	380 (46.5)	394 (47.7)	35 (4.3)	51 (6.2)	N/A	N/A	N/A	N/A	SAPIEN XT/ SAPIEN 3/ CoreValve/ CoreValve Evolut R/ Lotus/ Portico/ Acurate Neo (SEV/BEV)
Collet et al., 2022	82.3±6.4	81.6±6.1	Female 391 (52.1)	Female 405 (54.1)	27.33 (5.16)	27.52 (5.45)	5.1±5.4	5.1±5.0	214 (28.5)	221 (29.5)	601 (80.0)	606 (80.9)	284 (37.8)	292 (39.0)	89 (11.9)	78 (10.4)	90 (12.0)	83 (11.1)	224 (29.82)	240 (32.04)	SEV/BEV

[ATLANTIS]																				
Naser et al, 2023	80 (11)	81 (9)	Male 161 (59)	Male 63 (41)	28.8 (5.75)	28.3 (5.99)	8.5 (4.7)	8.2 (6.3)	59 (39)	94 (34)	N/A	N/A	N/A	N/A	14 (9)	53 (8)	47 (31)	70 (26)	85	276	SAPIEN XT and older/ SAPIEN S3/ Sapien3Ultra (BEV)

* The study by Rogers et al. (2021) is a 30-day follow-up, while the study by Merdler et al. (2023) is a 24-month follow-up survey of LRT 2.0, both using the same study population. All baseline characteristics were obtained from the Rogers et al. study.

Abbreviations: BEV: balloon-expandable valve, CHF: congestive heart failure, DM: diabetes mellitus, HTN: hypertension, PCI: percutaneous coronary intervention, SEV: self-expandable valve.

Supplemental Table 3. Eligibility criteria and number of events for each outcome in included studies

Study	Inclusion Criteria	Exclusion Criteria	Assessment of Outcomes	All-Cause Mortality N. of events		Cardiovascular Mortality N. of events		Stroke N. of events		Myocardial Infarction N. of events		Life-threatening or Major Bleeding N. of events		Valve Dysfunction/Thrombosis N. of events	
				Arm 1	Arm 2	Arm 1	Arm 2	Arm 1	Arm 2	Arm 1	Arm 2	Arm 1	Arm 2	Arm 1	Arm 2
**SAPT vs. DAPT**															
Ussia et al., 2011	All patients who fulfilled the clinical and anatomic criteria for TAVR	-Previous PCI or ACS requiring DAPT<br>-The need for OAC therapy<br>-Allergy or intolerance to any of the trial drugs	VARC	5	4	0	1	3	2	0	1	3	4	N/A	N/A
Stabile et al., 2014 [SAT-TAVR]	All patients with:<br>- Severe AS<br>- Cardiac symptoms: NYHA Functional Class ≥II, Syncope.<br>- High surgical risk: Predicted risk of operative mortality ≥15% or STS score ≥10	- Aortic annulus diameter < 18 mm or >25 mm<br>- Aortic dissection or iliac-femoral dimensions or disease precluding safe sheath insertion<br>- Untreated CAD requiring revascularization<br>- Severe AR or MR (>3+) or prosthetic valve (any location)<br>- Acute MI within 1 month<br>- GIB within 3 months<br>- CVA or TIA within 6 months<br>- Any cardiac procedure, other than BAV, within 1 month or within 6 months for DES<br>- Indication for OAC therapy<br>- Aspirin intolerance/allergy<br>- Thienopiridine intolerance/allergy	VARC	3	3	2	1	2	1	N/A	N/A	4	5	N/A	N/A
D'Ascenzo et al, 2017	All patients who underwent TAVR using BEV	Indication for OAC therapy	VARC-2	157	163	N/A	N/A	4	9	N/A	N/A	8	24	17	18
Ichibori et al., 2017	All patients who underwent TAVR using BEV	Indication for OAC therapy	VARC-2	3	3	N/A	N/A	4	4	1	2	2	8	N/A	N/A
Rodés-Cabau et al., 2017 [ARTE]	All patients with clinical indications for TAVR with BEV	-Need for chronic OAC treatment<br>- Major bleeding within the 3 months before the TAVR<br>- Prior intracranial bleeding<br>-DES implantation within the year before the TAVR<br>- Allergy to Clopidogrel and/or Aspirin	VARC-2	4	7	N/A	N/A	1	3	1	4	4	12	N/A	N/A
Brouwer et al., 2020 [Cohort A POPular TAVR]	All patients who underwent TAVR	-Long-term indication for OAC<br>- DES implantation within 3 months prior to TAVR<br>- Bare-metal stent implantation within 1 month prior to TAVR	VARC-2<br>BARC<br>TIMI<br>GUSTO	21	19	14	13	17	19	4	6	17	36	3	1

		- Allergy, intolerance or contraindication to Aspirin or Clopidogrel													
**SAPT vs. OAC+SAPT**															
Merdler et al. 2023 [LRT 2.0]*	All low-risk patients who underwent TAVR	- Not low risk<br>- Bicuspid aortic valve<br>- Patient chose surgery<br>- Patient chose not to participate in research<br>- Transfemoral access not feasible	VARC-2 BARC	1	1	N/A	N/A	N/A	N/A	N/A	N/A	N/A	N/A	N/A	N/A
**DAPT vs. OAC**															
Park et al., 2022 [ADAPT-TAVR]	Patients aged ≥18 with symptomatic AS who underwent TAVR	- Indication for Anticoagulation<br>- Any absolute indication for DAPT<br>- Severe renal insufficiency<br>- Prohibiting CT imaging (estimated glomerular<br>filtration rate <30 mL per minute per 1.73 m2 of body surface area)	VARC	2	3	0	3	2	2	3	1	3	6	N/A	N/A
**DAPT vs. OAC+SAPT**															
Dangas et al., 2020 [GALILEO]	Men and women 18 years of age or older who underwent TAVR	-Any established indication for long-term Anticoagulation<br>-Indication for Dual Antiplatelet therapy	VARC	38	64	27	35	25	30	17	23	15	30	N/A	N/A
Collet et al., 2022 [ATLANTIS] Stratum 2	All patients who underwent TAVR	Atrial Fibrillation	VARC-2	14	35	9	21	N/A	N/A	N/A	N/A	49	78	N/A	N/A
Naser et al, 2023	All patients who underwent TAVR	-Atrial Fibrillation<br>-Ischemic stroke within 30 days before or after TAVR	VARC-2	78	112	N/A	N/A	6	15	N/A	N/A	N/A	N/A	12	3

* The study by Rogers et al. (2021) is a 30-day follow-up, while the study by Merdler et al. (2023) is a 24-month follow-up survey of LRT 2.0, both using the same study population. All baseline characteristics were obtained from the Rogers et al. study.

Abbreviations: ACS: acute coronary syndrome, AR: aortic regurgitation, BARC: Bleeding Academic Research Consortium, BAV: balloon aortic valvuloplasty, CAD: coronary artery disease, CVA: cerebrovascular accident, DAPT: dual antiplatelet therapy, DES: drug-eluting stent, GIB: upper gastrointestinal bleeding, GUSTO: Global Utilization of Streptokinase and Tissue Plasminogen Activator for Occluded Arteries, MI: myocardial infarction, MR: mitral regurgitation, OAC: oral anticoagulation, PCI: percutaneous coronary intervention, SAPT: single antiplatelet therapy, TAVR: transcatheter aortic valve replacement, TIMI: Thrombolysis in Myocardial Infarction, TIA: transient ischemic attack, VARC: Valve Academic Research Consortium.

Supplemental Table 4. Publication bias assessment using Egger's and Begg's tests

	Number of Studies	Egger's Test P-value	Begg's Test P-value for SAPT vs. DAPT
**All-Cause Mortality**	13	0.951	0.652
**Cardiovascular Mortality**	8	0.331	0.497
**Total Bleeding**	9	0.717	0.624
**Major or Life-threatening Bleeding**	12	0.343	0.652
**Myocardial Infarction**	7	0.463	0.497
**Stroke**	10	0.988	0.348

Supplemental Table 5. PRISMA 2020 checklist items

Section and Topic	Item #	Checklist item	Location where item is reported
TITLE			
Title	1	Identify the report as a systematic review, meta-analysis, or both.	1
ABSTRACT			
Abstract	2	Provide a structured summary including, as applicable: background; objectives; data sources; study eligibility criteria, participants, and interventions; study appraisal and synthesis methods; results; limitations; conclusions and implications of key findings; systematic review registration number.	1
INTRODUCTION			
Rationale	3	Describe the rationale for the review in the context of existing knowledge.	1
Objectives	4	Provide an explicit statement of questions being addressed with reference to participants, interventions, comparisons, outcomes, and study design (PICOS).	1
METHODS			
Protocol and registration	5	Indicate if a review protocol exists, if and where it can be accessed (e.g., Web address), and, if available, provide registration information including registration number.	2
Eligibility criteria	6	Specify the inclusion and exclusion criteria for the review and how studies were grouped for the syntheses.	2
Information sources	7	Describe all information sources (e.g., databases with dates of coverage, contact with study authors to identify additional studies) in the search and date last searched.	3
Search strategy	8	Present full electronic search strategy for at least one database, including any limits used, such that it could be repeated.	3
Selection process	9	Specify the methods used to decide whether a study met the inclusion criteria of the review, including how many reviewers screened each record and each report retrieved, whether they worked independently, and if applicable, details of automation tools used in the process.	4
Data collection process	10	Specify the methods used to collect data from reports, including how many reviewers collected data from each report, whether they worked independently, any processes for obtaining or confirming data from study investigators, and if applicable, details of automation tools used in the process.	4
Data items	11a	List and define all outcomes for which data were sought. Specify whether all results that were compatible with each outcome domain in each study were sought (e.g. for all measures, time points, analyses), and if not, the methods used to decide which results to collect.	4
	11b	List and define all other variables for which data were sought (e.g. participant and intervention characteristics, funding sources). Describe any assumptions made about any missing or unclear information.	4
Study risk of bias assessment	12	Specify the methods used to assess risk of bias in the included studies, including details of the tool(s) used, how many reviewers assessed each study and whether they worked independently, and if applicable, details of automation tools used in the process.	5
Effect measures	13	Specify for each outcome the effect measure(s) (e.g. risk ratio, mean difference) used in the synthesis or presentation of results.	5
Synthesis methods	14	Describe the methods of handling data and combining results of studies, if done, including measures of consistency (e.g., I^2) for each meta-analysis.	5
Reporting bias assessment	15	Specify any assessment of risk of bias that may affect the cumulative evidence (e.g., publication bias, selective reporting within studies).	5
Additional analyses	16	Describe methods of additional analyses (e.g., sensitivity or subgroup analyses, meta-regression), if done, indicating which were pre-specified.	5
RESULTS			

Section and Topic	Item #	Checklist item	Location where item is reported
Study selection	17	Give the number of studies screened, assessed for eligibility, and included in the review, with reasons for exclusions at each stage, ideally with a flow diagram.	6 + figure 1
Study characteristics	18	Cite each included study and present its characteristics.	6 and table 1 + supplemental table 1
Risk of bias in studies	19	Present assessments of risk of bias for each included study.	9 + Supplemental figures 4 and 5
Results of individual studies	20	For all outcomes, present, for each study: (a) summary statistics for each group (where appropriate) and (b) an effect estimate and its precision (e.g. confidence/credible interval), ideally using structured tables or plots.	Figures 3 and 4 + supplemental figures 1 and 2
Results of syntheses	21	Present results of each meta-analysis done, including confidence intervals and measures of consistency.	
			6 and 7
Reporting biases	22	Present assessments of risk of bias due to missing results (arising from reporting biases) for each synthesis assessed.	9 + supplemental figures 4 and 5
Additional analysis	23	Give results of additional analyses, if done (e.g., sensitivity or subgroup analyses, meta-regression [see Item 16]).	8 and 9 + supplemental figures 2, 3 and 6
DISCUSSION			
Discussion	24a	Provide a general interpretation of the results in the context of other evidence.	10-13
	24b	Discuss any limitations of the evidence included in the review.	13
	24c	Discuss any limitations of the review processes used.	13
	24d	Discuss the implications of the results for practice, policy, and future research.	13 and 14
OTHER INFORMATION			
Support	25	Describe sources of financial or non-financial support for the review, and the role of the funders or sponsors in the review.	1
Competing interests	26	Declare any competing interests of review authors.	2
Availability of data, code, and other materials	27	Report which of the following are publicly available and where they can be found: template data collection forms; data extracted from included studies; data used for all analyses; analytic code; any other materials used in the review.	2
